# Supplementary material for: Learning interpretable network dynamics via universal neural symbolic regression
Source: Nat Commun. 2025 Jul 6;16:6226. doi: 10.1038/s41467-025-61575-7 (PMC12228748; doi:10.1038/s41467-025-61575-7)
Supplement: Supplementary file 1 — Supplementary Information [file 41467_2025_61575_MOESM1_ESM.pdf]

# SUPPLEMENTARY INFORMATION for *Learning Interpretable Network Dynamics via Universal Neural Symbolic Regression*


**I Network Dynamics and Topologies** **2**
- A Network dynamics . . . . . 2
- B Network topologies . . . . . 3
- C Initial conditions, sampling intervals, and end time of inferring and predicting 5
- D Description of mask settings . . . . . 5

**II Details of Neural Architectures in Signal Decoupling** **6**

**III Training Set for Transformer** **7**

**IV More Results of Inferring One-Dimensional Network Dynamics** **7**
- A Comparison of discovered equations . . . . . 7
- B More performance comparison . . . . . 10

**V Robustness Evaluation** **11**
- A Noisy observations and missing topology . . . . . 11
- B Comparison of different sampling frequencies . . . . . 13
- C Comparison of difference methods . . . . . 14

**VI An Ablation Experiment to Evaluate the Effectiveness of the Signal Decoupling Architecture Design** **15**

**VII Inferring Higher-Order Network Dynamics with Multi-Node Interaction** **16**

**VIII Multi-Dimensional and Heterogeneous Network Dynamics** **17**
- A FitzHugh-Nagumo dynamics . . . . . 17
- B Predator-prey system . . . . . 18

**IX Chaotic Networks Dynamics** **19**
- A Lorenz system . . . . . 19
- B Rössler system . . . . . 20

**X Empirical Systems** **22**
- A Real-world global epidemic transmission . . . . . 22
- B Pedestrian dynamics . . . . . 25

**XI Pseudo-code of the LLC** **25**

# I Network Dynamics and Topologies

## A Network dynamics

We study six representative one-dimensional homogeneous network dynamics spanning the fields of biology, ecology, epidemics, and neuroscience.

- Biochemical Dynamics (Bio): Biochemical processes within living cells are mediated by protein-protein interactions in which proteins bind to form protein complexes [1]. Its dynamics of biochemical reactions can be formulated as: $\dot{X}_i(t) = F_i + B_i X_i(t) + \sum_{j=1}^{N} A_{i,j} X_i(t) X_j(t)$, where $X_i(t)$ is the concentration of protein $i$ at time $t$, $A_{i,j}$ is the effective rate constant of interaction between proteins $i$ and $j$, and $F_i$ and $B_i$ denote the average influx rate and degradation rate of proteins $i$, respectively. For simplicity, we set $F_i = 1$ and $B_i = -1$.
- Gene Regulatory Dynamics (Gene): The dynamics of gene regulatory networks can be described by the Michaelis-Menten equation [2, 3], given by $\dot{X}_i(t) = -B_i X_i(t)^f + \sum_{j=1}^{N} A_{i,j} \frac{X_j(t)^h}{X_j(t)^h + 1}$. The node state $X_i(t)$ is the expression level of gene $i$. Parameter $B_i$ controls the decay rate. When $f = 1$, the first term on the right-hand side represents degradation, where the expression level of gene $i$ decreases over time. When $f = 2$, it describes dimerization corresponding to two or two of the same molecules together to form dimers. The second term captures genetic activation, where $h \geq 0$ represents the Hill coefficient, quantifying the saturation rate affected by neighboring nodes. We set $B_i = 2$, $f = 1$, and $h = 2$ here.
- Mutualistic Interaction Dynamics (MI): The dynamics of mutualistic interactions between species in ecology can be described by $\dot{X}_i(t) = b_i + X_i(t)(1 - \frac{X_i(t)}{k_i})(\frac{X_i(t)}{c_i} - 1) + \sum_{j=1}^{N} A_{i,j} \frac{X_i(t) X_j(t)}{d_i + e_i X_i(t) + h_i X_j(t)}$. The abundance $X_i(t)$ of a captured species in a mutualistic differential equation system [4] consists of an afferent migration term $b_i$, a logical increase in population capacity $k_i$, an Allee effect with a cold starting threshold $c_i$, and a mutualistic interaction term with the interaction network $A$. The parameters are set as $b_i = 1$, $k_i = 5$, $c_i = 1$, $d_i = 5$, $e_i = 0.9$, and $h_i = 0.1$.
- Lotka-Volterra Model (LV): The Lotka-Volterra model (LV) [5] describes the population dynamics of species in competition: $\dot{X}_i(t) = X_i(t)(\alpha_i - \theta_i X_i(t)) - \sum_{j=1}^{N} A_{i,j} X_i(t) X_j(t)$. Similar to mutualistic dynamics, the node state $X_i(t)$ represents the population size of species $i$, the growth parameters of species $i$ denoted by $\alpha_i, \theta_i$, are both positive constants. In experiments, we sample $\alpha_i, \theta_i$ from a uniform distribution within the range $[0.5, 1.5]$. For specific simulations, we set the values of $\alpha_i$ and $\theta_i$ to 0.5 and 1, respectively.
- Neural Dynamics (Neur): The firing rate of a neuron can be described by the Wilson-Cowan [6] model as $\dot{X}_i(t) = -X_i(t) + \sum_{j}^{N} A_{i,j}(1 + exp(-\tau(X_j(t) - \mu)))^{-1}$. In this model, $X_i(t)$ represents the activity level of neuron $i$, while the parameters $\tau$ and $\mu$ determine the slope and threshold of the neural activation function, respectively. For our experiments, we set $\tau = 1$ and $\mu = 1$.
- Epidemic Dynamics (Epi): Epidemic dynamics can be used to describe the outbreak of infectious diseases [7] as: $\dot{X}_i(t) = -\delta_i X_i(t) + \sum_{j=1}^{N} A_{i,j}(1 - X_i(t)) X_j(t)$. In this model, each node can represent an individual, where the node state $X_i \in [0, 1]$ corresponds to the infection probability of node $i$. The parameter $\delta_i$ represents the rate at which individuals recover from infection, which is set to 1.0 in our experiments.
- Kuramato Dynamics (Kura): Kuramoto model [8] is a classical mathematical model to study the synchronization phenomenon of coupled oscillators. $\dot{X}_i(t) = \omega_i + \epsilon \sum_{j=1}^{N} A_{i,j} sin(X_j(t) - X_i(t))$, where $X_i(t)$ denotes the phase (angle over time) of the $i$-th oscillator. Let $\omega_i$ denote the natural frequency of the $i$-th oscillator, which follows a Gaussian distribution. $\epsilon$ represents the coupling strength, which controls the strength of the interaction between the oscillators.
- FitzHugh-Nagumo model (FHN): FHN is a neuron model that describes the excitatory behavior of neurons. As a simplified version of the Hodgkin-Huxley model, it is primarily used to study neuron action potentials.$\dot{X}_{i1}(t) = X_{i,1}(t) - X_{i,1}^3(t) - X_{i,2}(t) +$

$\epsilon \sum_{j=1}^{N} A_{ij} \frac{(X_j(t)-X_i(t))}{K_{in}}$, $\dot{X}_{i2}(t) = a + bX_{i,1}(t) + cX_{i,2}(t)$ The first component $X_{i,1}$ denotes the membrane potential containing the self and interaction dynamics, $K_{in}$ is the in-degree of neuron $i$ (denoting the number of afferent connections to node $i$), and $\epsilon = 1$. The second component $X_{i,2}$ denotes the recovery variable. We set $a = 0.28, b = 0.5, c = -0.04$.

- Predator-Prey model (PP): This is a heterogeneous system [9], where the node state represents the position of each individual. Nodes are classified into two roles: a single predator ($i = 0$, $\dot{X}_0(t) = \frac{X_j(t)-X_0(t)}{|X_j(t)-X_0(t)|^2}$) and multiple preys ($i > 0$, $\dot{X}_i(t) = b\frac{X_i(t)-X_0(t)}{|X_i(t)-X_0(t)|^2} + \frac{1}{N}\sum_{j=1}^{N}\left(\frac{X_j(t)-X_i(t)}{|X_j(t)-X_i(t)|^2} + a(X_j(t) - X_i(t))\right)$), leading to three types of pairwise interactions: predator-prey, prey-predator, and prey-prey. The interactions between prey are modeled to exhibit paired short-range repulsion and long-range attraction. we set $a, b$, and $c$ to 1.0, 0.2, and 0.7.
- Rössler system: The Rössler system is a set of ordinary differential equations describing chaotic dynamics. $\dot{X}_{i1}(t) = -X_{i2}(t) - X_{i3}(t) + \epsilon\sum_{j=1}^{N} A_{ij}(X_{j1}(t) - X_{i1}(t))$, $\dot{X}_{i2}(t) = X_{i1}(t) + aX_{i2}(t)$, $\dot{X}_{i3}(t) = b + X_{i3}(t)(X_{i1}(t) - c)$, where $\epsilon = 0.15, a = 0.2, b = 0.2$ and $c = 5.7$ are system parameters. The first dimension shows that the change of $X_{i1}$ is driven by the negative coupling of $X_{i2}$ and $X_{i3}$, which is similar to the damping effect. The second dimension shows the variation of $X_{i2}$ consisting of the linear actuation of $X_{i1}$ and its own feedback with the coefficient $a$ controlling the strength of the feedback. The third dimension is the key nonlinear term. The constant $b$ provides the base growth rate for $X_{i3}$, while $X_{i3}(X_{i1} - c)$ introduces a threshold mechanism: when $X_{i1} > c$, $X_{i3}$ grows exponentially; and vice versa. This "switch" behavior leads to the stretching and folding of phase space trajectories, which is the source of chaos.
- Lorenz system: $\dot{X}_{i1}(t) = a(X_{i2}(t) - X_{i1}(t)) + \epsilon\sum_{j=1}^{N} A_{ij}(X_{j1}(t) - X_{i1}(t))$, $\dot{X}_{i2}(t) = X_{i1}(t)(r - X_{i3}(t)) - X_{i2}(t)$, $\dot{X}_{i3}(t) = X_{i1}(t)X_{i2}(t) - bX_{i3}(t)$, where $a = 10, \epsilon = 0.05$, $r = 28$ and $b = 10/3$ are system parameters. The first dimension indicates that the change in $X_{i1}$ is driven by the difference between $X_{i2}$ and $X_{i1}$, the coefficient $a$ (Prandtl number) controls the transfer rate of the difference. The second dimension contains two key roles: $X_{i1}(r - X_{i3})$ is the nonlinear coupling term that introduces the feedback from $X_{i3}$ to $X_{i1}$, and $X_{i2}$ is the damping term, which suppresses the growth of $x_{i2}$. The parameter $r$ (Rayleigh number) determines the stability of the system. The third dimension $X_{i1}X_{i2} - bX_{i3}$ combines energy conservation and nonlinearity. The term $X_{i1}X_{i2}$ denotes the synergy between $X_{i1}$ and $X_{i2}$ to drive growth in $X_{i3}$.

In fact, the ratio of nonlinear to linear coupling in our testing systems is 11:4. The specific description is shown in Supplementary Table 1. The experimental results in our work indicate that the proposed LLC consistently recovers the network dynamics equations accurately, regardless of whether the system exhibits linear or nonlinear coupling.

## B Network topologies

Network topology plays a crucial role in the evolution of node states. In this work, we investigate four network topologies: the Erdős-Rényi (ER) network [10], the Barabási-Albert (BA) scale-free network [11], and two empirical networks, namely C. elegans [12] and Drosophila [13]. Below, we describe the unique process used to generate each of these topologies.

- Synthetic networks: (1) Erdős-Rényi (ER) network [10], also known as a random network, is characterized by node degrees drawn from a Poisson distribution with mean degree $k = (n-1)p$, where $p$ is the probability of edge creation. (2) Barabási-Albert (BA) network [11] is characterized by a power-law degree distribution. The network topology is constructed using a preferential attachment mechanism: starting with an initial set of nodes, new nodes are iteratively introduced. Each new node connects to existing nodes with a probability proportional to their degree.
- Empirical networks: (3) the neural connectome of the C.elegans [12], the nematode worm Caenorhabditis elegans, consists of the 279 neurons between 2990 synaptic connections on the cellular network. (4) Drosophila [13], the cellular connectome of the mushroom body region in Drosophila melanogaster (fruit fly), which includes neurons that project

Supplementary Table 1: Description of coupling term types

| **Coupling term type** | **Specific classification** | **Network dynamics** | **Governing equations** |
|---|---|---|---|
| Linear | Linear | FHN | $\dot{X}_{i,1}(t) = X_{i1} - X_{i1}^2 - X_{i2} - \epsilon \sum_{j=1}^{N} A_{ij} \frac{(X_j - X_i)}{k_{in}}$ |
| | | Rossler | $\dot{X}_{i,1}(t) = -X_{i2} - X_{i3} + \epsilon \sum_{j=1}^{N} A_{ij}(X_j - X_i)$ |
| | | Lorenz | $\dot{x}_{i,1}(t) = 10(X_{i2} - X_{i1}) + \epsilon \sum_{j=1}^{N} A_{ij}(X_j - X_i)$ |
| | | Heat | $\dot{X}_i(t) = 0.5 \sum_{j=1}^{N} A_{ij}(X_j - X_i)$ |
| Nonlinear | Multiplicative coupling terms | Bio | $\dot{X}_i(t) = 1 - x_i + \sum_{j=1}^{N} A_{ij}(X_i * X_j)$ |
| | | Gene | $\dot{X}_i(t) = -2.000 x_i + \sum_{j=1}^{N} A_{ij} \frac{X_j^2}{1+X_j^2}$ |
| | | LV | $\dot{X}_i(t) = 0.5 X_i - X_i^2 - \sum_{j=1}^{N} A_{ij} X_j X_i$ |
| | | Epi | $\dot{X}_i(t) = -x_i + \sum_{j=1}^{N} A_{ij} X_j (1 - X_i)$ |
| | | High-order2 | $\dot{X}_i(t) = -0.3 X_i + \sum_{jk} A_{ijk} X_j X_k (1 - \lvert X_i \rvert)$ |
| | Asymmetric/directional coupling | MI | $\dot{X}_i(t) = 1 - 0.2 X_i^3 + 1.2 X_i^2 - X_i + \sum_{j=1}^{N} A_{ij} \frac{X_i X_j}{5 + 0.9 X_i + 0.1 X_j}$ |
| | | Predator-prey | $\dot{X}_0(t) = \frac{c}{N} \sum_{j=1}^{N} \frac{X_j - X_0}{\lvert X_j - X_0 \rvert^2}, \dot{X}_{i>0}(t) = b \frac{X_i - X_0}{\lvert X_i - X_0 \rvert^2} + \frac{1}{N} \sum_{j=1}^{N} \left( \frac{X_j - X_i}{\lvert X_j - X_i \rvert^2} + a(X_j - X_i) \right)$ |
| | Nonlinear excitation coupling | Kuramoto | $\dot{X}_i(t) = w_i + \sum_{j=1}^{N} A_{ij} \sin(X_j - X_i)$ |
| | | Neural | $\dot{X}_i(t) = -X_i + \sum_{j=1}^{N} A_{ij} \frac{1}{1 + \exp(-(X_j - 1))}$ |
| | | High-order1 | $\dot{X}_i(t) = \sum_{j=1}^{N} A_{ijk} \exp(l \lvert X_k - X_j \rvert) [(X_j - X_i)(X_k - X_i)]$ |
| | | Real datasets | Showing obvious nonlinearity, and the inferred coupling terms contain $\frac{X_i X_j}{a + b X_i + c X_j}, \frac{b X_j}{e^{c X_j / X_i}}, \frac{a X_i}{X_j + b}$ and so on |

their axons into bundles similar to paired mushrooms, can be accessed through https://neuprint-examples.janelia.org/.

The ER network is primarily used to generate data for comprehensive comparative experiments on one-dimensional homogeneous network dynamics, while the BA network and two empirical networks are mainly employed for data generation in the context of inferring multi-dimensional, heterogeneous, and chaotic network dynamics.

Supplementary Table 2: Explanations of network extension and supplementation in different experimental scenarios.

| **Scenario** | **Topology** |
|---|---|
| Bio | ER, Poisson distribution, No Scale-free |
| Gene | ER, Poisson distribution, No Scale-free |
| MI | ER, Poisson distribution, No Scale-free |
| LV | ER, Poisson distribution, No Scale-free |
| Neural | ER, Poisson distribution, No Scale-free |
| Epi | ER, Poisson distribution, No Scale-free |
| Multi-dimensional Dynamics (FHN) | Barabasi-Albert, Power-law distribution, Scale-free<br>Drosophila, Power-law distribution, Scale-free<br>C.elegans, Power-law distribution, Scale-free |
| Predator-prey network dynamics | Fully connected, No Scale-free |
| Rosssler dynamics | BA, Power-law distribution, Scale-free |
| Pedestrians dynamics | Dynamic topology, No Scale-free |
| COVID-19 dynamics | BA, Power-law distribution, Scale-free |
| Robustness valuation experiment | BA, Power-law distribution, Scale-free |

# C Initial conditions, sampling intervals, and end time of inferring and predicting

To ensure the reproducibility of our findings, we provide a comprehensive list of parameter settings on the initial conditions, sampling intervals, and end time of inferring and predicting, where let $T$ and $T_{end}$ denote the end time of inference and end time of prediction, respectively. If not explicitly stated, the number of nodes ($N$) is set to 100. The simulations encompass dynamics from time $t = 0$ to $T$ or $T_{end}$ with a step-size $\delta t$. Supplementary Table 3 presents the initial conditions, sampling intervals, and end time of inferring and predicting used to simulate network dynamics data.

Supplementary Table 3: Initial conditions, sampling intervals, and end time of inferring ($T$) and predicting ($T_{end}$) used to simulate network dynamics data.

| Dynamics | Initial Condition ($t = 0$) | $\delta t$ | $T$ | $T_{end}$ |
|---|---|---|---|---|
| Bio | Uniform distribution(0,2) | 0.0001 | 0.1 | 0.5 |
| Gene | Uniform distribution(0,2) | 0.01 | 5 | 10 |
| MI | Uniform distribution(0,2) | 0.001 | 1 | 5 |
| LV | Uniform distribution(0,5) | 0.0001 | 0.1 | 0.5 |
| Neur | Uniform distribution(0,2) | 0.01 | 5 | 10 |
| Epi | Uniform distribution(0,1) | 0.001 | 1 | 5 |

# D Description of mask settings

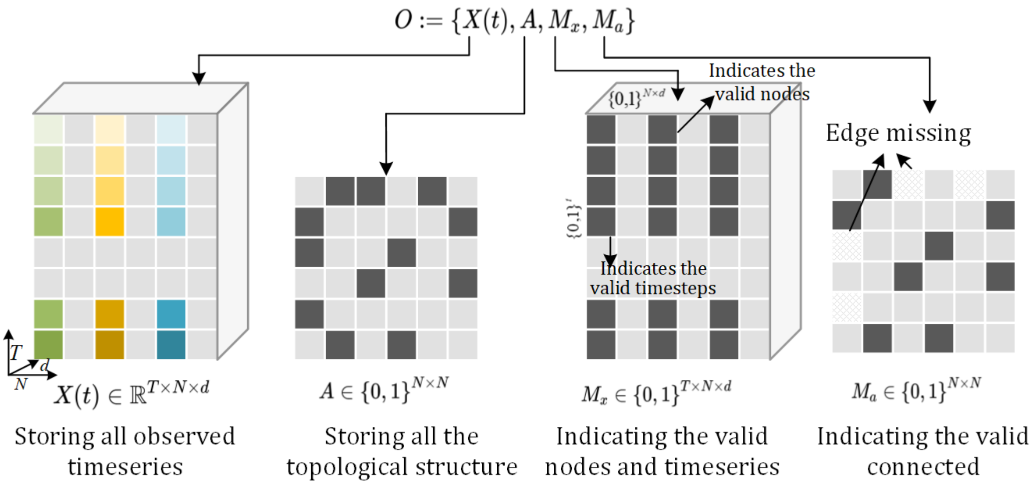


Supplementary Figure 1: An illustration for $O = X(t), A, M_x, M_a$. $X(t) \in \mathbb{R}^{T\times N}$ stores all observed time series and the mask has the same shape as $X(t)$, indicating which nodes has been observed (its values on the corresponding position is set to 1,otherwise it is 0). $A \in 0, 1^{N\times N}$ represents the topological structure at time. $M_a$ and $A$ have the same shape, indicating that the edge is valid during the experiment (its values on the corresponding position is set to 1,otherwise it is 0).

The introduction of the observation mask $M_x$ and the topology mask $M_a$ during the training process can effectively differentiate between real observations and missing data. By leveraging the known node topology structure, these masks help reduce the uncertainty caused by missing data or an incomplete structure. This leads to more accurate gradient calculations, thereby improving training efficiency and convergence speed. In addition, this mechanism enhances the robustness and generalization ability of the model, ensuring that

it can still maintain high prediction performance and adaptability in the face of irregular, partially missing or unknown structure data. Supplementary Figure 1 gives a schematic illustration of the interpretation for dimension. Specific mask settings for different scenarios are listed in Supplementary Table 4 ($M_x(t, :, :) = 0$ means that all nodes are completely missing observations at time $t$. $M_a(i, j) = 0, j \in \mathcal{N}(i)$ indicates that the topological information at node $i$ are completely missing. )

Supplementary Table 4: Mask Settings in Different scenarios.

| **Experiment scenarios** | **Settings for** $M_x$ | **Settings for** $M_a$ |
|---|---|---|
| One-dimensional network dynamics (Section 2.2) | $M_x(t, :, :) = \begin{cases} 0, & t \geq 0.5T \\ 1, & \text{otherwise} \end{cases}$ | $M_a(i, j) = 1$ |
| Multi-dimensional network dynamics (Section 2.3) | $M_x(t, :, :) = \begin{cases} 0, & t \geq 0.5T \\ 1, & \text{otherwise} \end{cases}$ | $M_a(:, :, t) = 1$ |
| Heterogeneous network dynamics (Section 2.3) | predator: $M_x(:, i, :) = \begin{cases} 1, & i = 0 \\ 0, & \text{otherwise} \end{cases}$ prey: $M_x(:, i, :) = \begin{cases} 1, & i \geq 1 \\ 0, & \text{otherwise} \end{cases}$ | predator: $M_a(i, j) = \begin{cases} 1, & i = 0 \land j \in i\text{'s neighbours} \\ 0, & \text{otherwise} \end{cases}$ prey: $M_a(i, j) = \begin{cases} 1, & i \geq 1 \land j \in i\text{'s neighbours} \\ 0, & \text{otherwise} \end{cases}$ |
| Chaotic systems (Section 2.4) | $M_x(:, :, :) = 1$ | $M_a(:, :) = 1$ |
| Empirical pedestrians dynamics (Section 2.5) | $M_x(:, :, :) = 1$ | $M_a(:, :) = 1$ |
| Empirical epidemic spread (Section 2.5) | Total:$M_x(:, :, :) = 1$ Each:$M_x(:, i, :) = \begin{cases} 0, & i \neq \text{target region} \\ 1, & \text{otherwise} \end{cases}$ | Total:$M_a(:, :) = 1$ Each:$M_a(i, j) = \begin{cases} 0, & i \neq \text{target region} \\ 1, & \text{otherwise} \end{cases}$ |
| Evaluation on robustness (Appendix D) | Noise state:$M_x(:, :, :) = 1$ Topology missing:$M_x(:, :, :) = 1$ | Noise state:$M_a(:, :) = 1$ Topology missing:$M_a(i, j) = \begin{cases} 1, & i \geq 1 \land j \in i\text{'s neighbours} \\ 0, & \text{otherwise} \end{cases}$ |

# II Details of Neural Architectures in Signal Decoupling

The detailed architectures of the neural networks $\psi^f$, $\psi^{g_0}$, $\psi^{g_1}$ and $\psi^{g_2}$ in $\hat{Q}^{(self)}$ and $\hat{Q}^{(inter)}$ are provided in Supplementary Table 5. They were trained using a random division

Supplementary Table 5: The detailed architectures of the neural networks $\psi^f$, $\psi^{g_0}$, $\psi^{g_1}$ and $\psi^{g_2}$ in $\hat{Q}^{(self)}$ and $\hat{Q}^{(inter)}$, where $d$ is the state dimension of the system.

| Neural networks | $\psi^f$ | $\psi^{g_0}$ | $\psi^{g_1}$ | $\psi^{g_2}$ |
|---|---|---|---|---|
| Layers | Linear($d$,50)<br>Rational<br>Linear(50, 50)<br>Rational | Linear(2$d$, 50)<br>Rational<br>Linear(50, 50)<br>Rational<br>Linear(50, 50)<br>Rational | Linear($d$,50)<br>Rational<br>Linear(50, 50)<br>Rational<br>Linear(50, 50)<br>Rational | Linear($d$, 50)<br>Rational<br>Linear(50, 50)<br>Rational<br>Linear(50, 50)<br>Rational |
| Readout layer | Linear(50, $d$) | Linear(50, $d$) | Linear(50, $d$) | Linear(50, $d$) |

of the timestamps into training and validation sets, with ratios of 0.8 and 0.2, respectively,

and trained for 1,000 epochs using the AdamW optimizer. The optimization process stops when the error on the validation set falls below a certain threshold, or it stops early if the multi-step validation error remains unchanged. The learning rate was searched in the range of [1e-3, 1e-2], the weight decay value was set to 0.001, and the hidden dimension was set to 50. Note that, we employ a smooth, trainable activation function derived from the rational function in Supplementary Table 5. There are practical and theoretical reasons for choosing rational functions. First, rational functions are computationally efficient, particularly because the polynomials connected by the multivariate operators are well suited for parallel computation. Second, rational functions can approximate a broader range of functions more efficiently and accurately than polynomials, especially those with singularities or sharp changes. The specific rational activation function can be formulated as:

$$R(X_i(t)) = \frac{P(X_i(t))}{Q(X_i(t))} = \frac{\sum_{i=0}^{r_P} a_i X_i(t)}{1 + |\sum_{j=0}^{r_Q} b_j X_j(t)|},$$

where $P$ and $Q$ represent two polynomials, the numerator and denominator respectively. $r_P$ and $r_Q$ are set to be 3 and 2, respectively. The specific values of a and b are set as: $a_0 = 1.1915, a_1 = 1.5957, a_2 = 0.5000, a_3 = 0.0218$; $b_0 = 2.3830, b_1 = 0.0000, b_2 = 1.0000$.

# III Training Set for Transformer

We randomly synthesize a large number of expressions as the training set for transformer model. Each randomly generated expression tree contains at most five non-leaf nodes, and its operators and leaf types are sampled from a weighted distribution. After executing the variable dependency rule, we convert the expression from prefix notation to infix notation, simplify it with Python package "Sympy", and insert constant placeholders for later regression. In fact, the specific details are consistent with the process of producing expressions in the paper [14].

After generating the training set, we further verified whether the items of expressions in the experimental network dynamics scenarios in this paper appear in the training set. We conducted a matching statistics on the overlap between the testing scenarios and the expressions in the training set. The results show that although some basic symbol forms such as $X_iX_j$, $X_i - X_j$ or $sin(X_i - X_j)$ may exist in the training data, the more complex composite forms did not appear in the training set. In particular, many typical evaluation systems, such as $\exp(l|X_k - X_j|) [(X_j - X_i) + (X_k - X_i)]$, $\frac{X_i^2}{X_i^2+1}$, $\frac{X_iX_i}{aX_i+bX_j+c}$, etc. exhibit complex or multivariate interactions that far exceed the simple symbolic expressions in training set. This also demonstrates that pre-trained models on a large number of equations may emerge to handle complex tasks.

To verify that overlap has nothing to do with the validity of this paper, we conduct comparative experiments with LLC+GP and GNN+GP, where neither method employs symbol converters during inference. The results (Supplementary Table 6) show that LLC+GP consistently maintains higher fidelity in recovering the underlying dynamics, especially on the more challenging benchmarks. This demonstrates that the advantages arise primarily from the decomposition strategy itself, rather than from any memorization by the pre-trained transformer.

# IV More Results of Inferring One-Dimensional Network Dynamics

## A Comparison of discovered equations

Supplementary Table 7 shows the discovered equations for each network dynamics scenario, demonstrating the capability of various methods to learn interpretable network dynamics. TPSINDy-$\mathcal{H}_B$, which includes only the basic operations, and TPSINDy-$\mathcal{H}_W$, which lacks the function terms of the ground truth, both struggle to infer the correct form of the equations for all dynamic systems. Although TPSINDy-$\mathcal{H}_N$ with a library containing the

Supplementary Table 6: Compares LLC+GP and GNN+GP in different scenarios

| Scenario | True | GNN+GP | LLC+GP | Ours |
|---|---|---|---|---|
| Bio | $\dot{X}_i(t) = 1 - X_i + \sum_{j=1}^{N} A_{ij}(X_i X_j)$ | $\dot{X}_i(t) = 1.130(1 - X_i) + \sum_{j=1}^{N} A_{ij}(X_i X_j)$ | $\dot{X}_i(t) = 0.979 - X_i + \sum_{j=1}^{N} A_{ij}(X_i X_j)$ | $\dot{X}_i(t) = 1 - X_i + \sum_{j=1}^{N} A_{ij}(X_i X_j)$ |
| LV | $\dot{X}_i(t) = (0.5 - X_i)X_i - \sum_{j=1}^{N} A_{ij} X_j X_i$ | $\dot{X}_i(t) = (0.486 - 0.998X_i) X_i - \sum_{j=1}^{N} A_{ij} X_j X_i$ | $\dot{X}_i(t) = (0.486 - 0.998X_i) X_i - \sum_{j=1}^{N} A_{ij} X_j X_i$ | $\dot{X}_i(t) = (0.5 - 1.002X_i) X_i - \sum_{j=1}^{N} A_{ij} X_j X_i$ |
| Epi | $\dot{X}_i(t) = -X_i + \sum_{j=1}^{N} A_{ij} X_j (1 - X_i)$ | $\dot{X}_i(t) = -1.000X_i + \sum_{j=1}^{N} A_{ij} X_j (1 - X_i)$ | $\dot{X}_i(t) = -1.023X_i + \sum_{j=1}^{N} A_{ij} 1.023 X_j (1 - X_i)$ | $\dot{X}_i(t) = -1.000X_i + \sum_{j=1}^{N} A_{ij} X_j (1 - X_i)$ |
| Gene | $\dot{X}_i(t) = -2.000X_i + \sum_{j=1}^{N} A_{ij} \frac{X_j^2}{1 + X_j^2}$ | $\dot{X}_i(t) = -1.000X_i + \sum_{j=1}^{N} A_{ij} \frac{X_j^2}{1 + X_j^2}$ | $\dot{X}_i(t) = -1.999X_i + \sum_{j=1}^{N} A_{ij} \frac{X_j^2}{1 + X_j^2}$ | $\dot{X}_i(t) = -2.000X_i + \sum_{j=1}^{N} A_{ij} \frac{X_j^2}{1 + X_j^2}$ |
| Neural | $\dot{X}_i(t) = -X_i + \sum_{j=1}^{N} A_{ij} \frac{1}{1 + e^{-(X_j - 1)}}$ | $\dot{X}_i(t) = -0.596X_i + \sum_{j=1}^{N} A_{ij}(0.237X_j + 0.262)$ | $\dot{X}_i(t) = -X_i + \sum_{j=1}^{N} A_{ij} \frac{e^{X_j}}{2.710 + e^{X_j}}$ | $\dot{X}_i(t) = -X_i + \sum_{j=1}^{N} A_{ij} \frac{e^{X_j}}{2.712 + e^{X_j}}$ |
| MI | $\dot{X}_i(t) = 1.2X_i^2 - 0.2X_i^3 - X_i + 1 + \sum_{j=1}^{N} A_{ij} \frac{X_i X_j}{5 + 0.9X_i + 0.1X_j}$ | $\dot{X}_i(t) = 0.722X_i^2 + 1 - 0.170X_i^3 + \sum_{j=1}^{N} A_{ij} (0.192X_j X_i + 0.004X_j)$ | $\dot{X}_i(t) = 0.456 + (0.197X_i^2 + 5.213)(0.521 - X_i) + \sum_{j=1}^{N} A_{ij} \frac{X_i X_j}{4.989 + 0.899X_i + 0.100X_j}$ | $\dot{X}_i(t) = X_i^2(1.20 - 0.20X_i) - X_i + 0.854 + \sum_{j=1}^{N} A_{ij} \frac{X_i X_j}{4.864 + 0.905X_i + 0.105X_j}$ |

same function terms as ground truth, and TPSINDy-$\mathcal{H}_R$ with sufficient basis functions can partially restore the terms of the equation, they still fails to accurately infer the coefficients. That is to say, improper basis library settings, stemming from a lack of prior knowledge, make it even more challenging for TPSINDy to correctly identify the dynamics equations.

On the contrary, the proposed LLC can directly and accurately infer the dynamics equations from the node states. Additionally, it can further enhance the TPSINDy. When the terms obtained through the LLC are used as the basis function terms for the TPSINDy, the sparse regression method can accurately identify the dynamics equations. This is supported by comparing the discovered equations of TPSINDy-$\mathcal{H}_N$ and LLC+TPSINDy.

Supplementary Table 7: Comparison of inferred equations of network dynamics.

| Methods | Bio | Gene | MI | LV | Neur | Epi |
|---|---|---|---|---|---|---|
| True | $\dot{X}_i = 1 - X_i + \sum_{j=1}^{N} A_{ij} X_i X_j$ | $\dot{X}_i = -2.000X_i + \sum_{j=1}^{N} A_{ij} \frac{X_j^2}{1+X_j^2}$ | $\dot{X}_i = 1 + X_i(1 - \frac{X_i}{5})(X_i - 1) \sum_{j=1}^{N} A_{ij} \frac{X_i X_j}{5+0.9X_i+0.1X_j}$ | $\dot{X}_i = 0.5X_i - X_i^2 - \sum_{j=1}^{N} A_{ij} X_j X_i$ | $\dot{X}_i = -X_i + \sum_{j=1}^{N} A_{ij} \frac{1}{1+e^{-(X_j-1)}}$ | $\dot{X}_i = -X_i + \sum_{j=1}^{N} A_{ij} X_j (1 - X_i)$ |
| TPSINDy$-\mathcal{H}_B$ | $\dot{X}_i = 0.004e^{X_i} + \sum_{j=1}^{N} A_{ij} 0.494 X_j X_i$ | $\dot{X}_i = -1.163X_i + \sum_{j=1}^{N} A_{ij} (2.008 + 0.026X_j)$ | $\dot{X}_i = -6.407 + \sum_{j=1}^{N} A_{ij} \frac{77.520}{X_i X_j}$ | $\dot{X}_i = -0.181X_i^2 - \sum_{j=1}^{N} A_{ij} 1.135 X_j X_i$ | $\dot{X}_i = -0.123X_i^2 + 8.352 + \sum_{j=1}^{N} A_{ij} 1.128 \sin(X_j)$ | $\dot{X}_i = -0.769X_i^3 + \sum_{j=1}^{N} A_{ij} 1.330 (X_j - X_i)$ |
| TPSINDy$-\mathcal{H}_N$ | $\dot{X}_i = 0.078X_i^3 + \sum_{j=1}^{N} A_{ij} 0.923 X_i X_j$ | $\dot{X}_i = 2.071 - 0.853X_i + \sum_{j=1}^{N} A_{ij} 0.050 \frac{X_j^2}{1+X_j^2}$ | $\dot{X}_i = 1.047X_i - 0.015X_i^3 + \sum_{j=1}^{N} A_{ij} (0.01X_j - 0.281 (X_j - X_i))$ | $\dot{X}_i = -0.894 - 0.084X_i^2 - \sum_{j=1}^{N} A_{ij} 1.42 X_j X_i$ | $\dot{X}_i = -4.278X_i + 28.343 + \sum_{j=1}^{N} A_{ij} \frac{-0.215}{1+e^{-(X_j-1)}}$ | $\dot{X}_i = 4.126 + \sum_{j=1}^{N} A_{ij} (-1.286X_j - 0.147X_j X_i)$ |
| TPSINDy$-\mathcal{H}_W$ | $\dot{X}_i = 2.125 \cos(X_i) + \frac{311.387}{e^{-(X_i-5)}} + \sum_{j=1}^{N} A_{ij} (\frac{3.151 X_i X_j}{X_j + 1} + \frac{0.590(X_j - X_i)}{K_{\text{in}}})$ | $\dot{X}_i = \frac{1.392X_i}{K_{\text{in}}} + \sum_{j=1}^{N} A_{ij} 1.204(X_j - X_i)/K_{\text{in}}$ | $\dot{X}_i = \frac{-81.872}{e^{(-(X_i-10))}} + 5.536 \sin(X_i) + 9.506 + \sum_{j=1}^{N} A_{ij} 0.002X_j$ | $\dot{X}_i = \frac{-682.033}{e^{(-(X_i-5))}} + \sum_{j}^{N} A_{ij} (1.499 \frac{0.471 X_i X_j}{K_{in}})$ | $\dot{X}_i = \frac{-2453.529}{e^{(-(X_i-10))}} + 15.621 - \sum_{j=1}^{N} A_{ij} (0.074X_j + 0.321 \frac{X_j - X_i}{K_{\text{in}}})$ | $\dot{X}_i = \frac{-0.605X^5}{1 + X_i^5} + \sum_{j=1}^{N} A_{ij} 0.127$ |
| TPSINDy$-\mathcal{H}_R$ | $\dot{X}_i = -0.005X_i^3 + \sum_{j=1}^{N} A_{ij} (0.859 X_j X_i + 0.025(X_j - X_i))$ | $\dot{X}_i = \frac{-2.154e11}{e^{-5(X_i-10)}} + \sum_{j=1}^{N} A_{ij} 0.165$ | $\dot{X}_i = \frac{-69.860}{e^{-(X_i-10)}} + 0.725 \cos(X_i) + \frac{27.055X_i}{K_{in}} + \sum_{j=1}^{N} A_{ij} 0$ | $\dot{X}_i = 0.290 - \sum_{j=1}^{N} A_{ij} 1.078 X_i X_j$ | $\dot{X}_i = -0.451X_i + 0.159 + \sum_{j=1}^{N} A_{ij} \frac{0.013}{1+e^{-(X_j-1)}}$ | $\dot{X}_i = -0.259X_i^3 + 0.138 + \sum_{j=1}^{N} A_{ij} \frac{-0.007(X_i X_j)^5}{1+(X_i X_j)^3}$ |
| GNN+GP | $\dot{X}_i = 1.130 (1 - X_i) + \sum_{j=1}^{N} A_{ij} X_i X_j$ | $\dot{X}_i = -1.000X_i + \sum_{j=1}^{N} A_{ij} \frac{X_j^2}{2+X_j^2}$ | $\dot{X}_i = 0.722X_i^2 - 0.170X_i^3 + 1 + \sum_{j=1}^{N} A_{ij} (0.192 X_j X_i + 0.004X_j)$ | $\dot{X}_i = 0.486X_i - 0.998X_i^2 - \sum_{j=1}^{N} A_{i,j} X_j X_i$ | $\dot{X}_i = -0.596X_i + \sum_{j=1}^{N} A_{ij} (0.237X_j + 0.262)$ | $\dot{X}_i = -1.000X_i + \sum_{j=1}^{N} A_{ij} X_j (1 - X_i)$ |
| LLC+TPSINDy | $\dot{X}_i = (1 - X_i) + \sum_{j=1}^{N} A_{i,j} X_i X_j$ | $\dot{X}_i = -2.000X_i + \sum_{j=1}^{N} A_{ij} \frac{X_j^2}{1+X_j^2}$ | $\dot{X}_i = 1.136X_i^2 - 0.197X_i^3 - 0.534X_i + \sum_{j=1}^{N} A_{ij} \frac{X_i X_j}{4.86+0.90X_i+0.10X_j}$ | $\dot{X}_i = 0.499X_i - 1.000X_i^2 - \sum_{j=1}^{N} A_{ij} 0.999 X_i X_j.$ | $\dot{X}_i = -X_i + \sum_{j=1}^{N} A_{ij} \frac{1}{1+e^{-(X_j-1)}}$ | $\dot{X}_i = -1.000X_i + \sum_{j=1}^{N} A_{ij} X_j (1 - X_i)$ |
| LLC | $\dot{X}_i = 1 - X_i + \sum_{j=1}^{N} A_{ij} X_i X_j$ | $\dot{X}_i = -2.000X_i + \sum_{j=1}^{N} A_{i,j} \frac{X_j^2}{1+X_j^2}$ | $\dot{X}_i = X_i^2 (1.20 - 0.20X_i) - X_i + 0.854 + \sum_{j=1}^{N} A_{ij} \frac{X_i X_j}{4.86+0.90X_i+0.10X_j}$ | $\dot{X}_i = 0.500X_i - 1.000X_i^2 - \sum_{j=1}^{N} A_{ij} X_j X_i$ | $\dot{X}_i = -X_i + \sum_{j=1}^{N} A_{ij} \frac{e^{X_j}}{2.712 + e^{X_j}}$ | $\dot{X}_i = -1.000X_i + \sum_{j=1}^{N} A_{ij} X_j (1 - X_i)$ |

Note: In order to save space, we overlook the time t when presenting the equations.

## B More performance comparison

In addition to the two indicators presented in the main text, namely the $R^2$ score and Recall, we introduce more performance comparison metrics here to provide a more comprehensive comparison.

- **Mean Relative Error.** MRE (Mean Relative Error) is the mean absolute difference between the predicted and true values, normalized by the absolute value of the true values. By accounting for the scale of the true values, it provides a consistent evaluation across datasets with different magnitudes, making it universally applicable. It can be calculated as:

$$MRE = \frac{1}{TN}\sum_{i=1}^{N}\sum_{t=1}^{T}|\frac{X_i(t) - \hat{X}_i(t)}{X_i(t)}|,$$

  where, $N$ and $T$ are the number of system nodes and the maximum prediction time, respectively. $\hat{X}_i(t)$ is the predicted state of node $i$ at time $t$, and $X_i(t)$ is the ground truth.
- **Mean Absolute Error.** MAE quantifies the mean absolute error between the predictions and the true values over all nodes and at all times, which can be calculated as

$$MAE = \frac{1}{TN}\sum_{i=1}^{N}\sum_{t=1}^{T}|X_i(t) - \hat{X}_i(t)|,$$

  where, $N$ and $T$ are the number of system nodes and the maximum prediction time, respectively. $\hat{X}_i(t)$ is the predicted state of node $i$ at time $t$, and $X_i(t)$ is the ground truth.
- **$\mathbf{L_2}$ error.** This error provides a measure of how closely the identified coefficients match the true coefficients in a relative sense, which can be seen as a quantitative indicator of whether the discovered equation is correct in form.

$$L_2 \text{ error} = \frac{|\xi_{\text{pre}} - \xi_{\text{True}}|_2}{|\xi_{\text{True}}|_2},$$

  where $\xi_{\text{pre}}$ represents the equation coefficients identified by a method and $\xi_{\text{True}}$ represents true coefficients.
- **Precision.** This statistic measures the percentage of correctly identified coefficients out of the total number of identified coefficients, which can be calculated as

$$P = \frac{|\xi_{\text{pre}} \odot \xi_{\text{true}}|_0}{|\xi_{\text{pre}}|_0},$$

  where $\odot$ represents the element-by-element product of two vectors, $|\cdot|_0$ denotes the number of non-zero elements in the vector.

Supplementary Figure 2 shows the performance comparison on the based above metrics. MRE and MAE values closer to 0 indicate smaller discrepancies between the predicted and true values. Compared to TPSINDy, the performance variations of our proposed methods across different dynamics are significantly smaller. Notably, the results of the proposed LLC are nearly optimal. Furthermore, in the comparison of equation forms, the best TPSINDy version can only identify ∼66.7% of the terms in the target equations, whereas our proposed LLC can identify nearly 100% of the terms.

Apart from performance comparison, Supplementary Figure 3 provides a visual example of the results produced by various methods in the MI scenario. Our LLC outperforms the others, which produces the predictive curve closest to the true values (see Supplementary Figure 3(a)). It is evident that the TPSINDy-$\mathcal{H}_W$ exhibits the worst performance, followed by the TPSINDy-$\mathcal{H}_R$. The TPSINDy-$\mathcal{H}_N$ approximates the true value well up to around 200 time steps, after which it gradually deviates from the ground truth and eventually and eventually maintains a stable distance around 600 time steps. In contrast, the node trajectories generated by the proposed LLC and LCC+TPSINDy consistently align

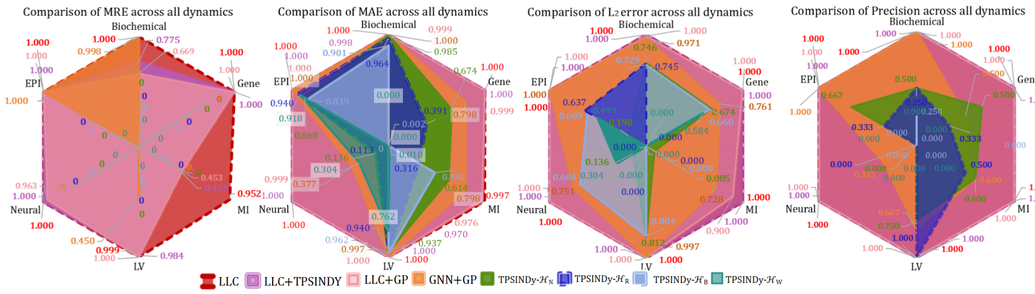


Supplementary Figure 2: Comparison of the accuracy on several metrics, including MRE (**a**), MAE (**b**), $L_2$ error (**c**), and Precision (**d**) for reconstructing dynamics from six scenarios, including Biochemical (Bio), Gene regulatory (Gene), Mutualistic Interaction (MI), Lotka-Volterra (LV), Neural (Neur), and Epidemic (Epi) dynamics. TPSINDy's results are highly dominated by its choice of function terms and our LLC significantly outperforms the comparative methods covering all network dynamics scenarios. For better visualization, MRE, MAE, and $L_2$ error are deflated transformed and mapped into [0, 1], and then the mapped value is subtracted by 1.

the true trajectories, thereby demonstrating the effectiveness of the proposed method compared to the TPSINDy. Supplementary Figure 3(b) shows the designed neural network fits the differential of node activity well, and effectively decomposes signal into self and interact components, as shown in Supplementary Figure 3(c-d). To visually illustrate the discrepancy between each node's predicted state and the ground truth, we employ the NED heatmap. It reveals that our LLC and LCC+TPSINDy exhibit minimal differences, while the others, although fitting well for some nodes, exhibit errors exceeding 1 and even reaching a maximum deviation of 3 in many other nodes, as shown in Supplementary Figure 3(e).

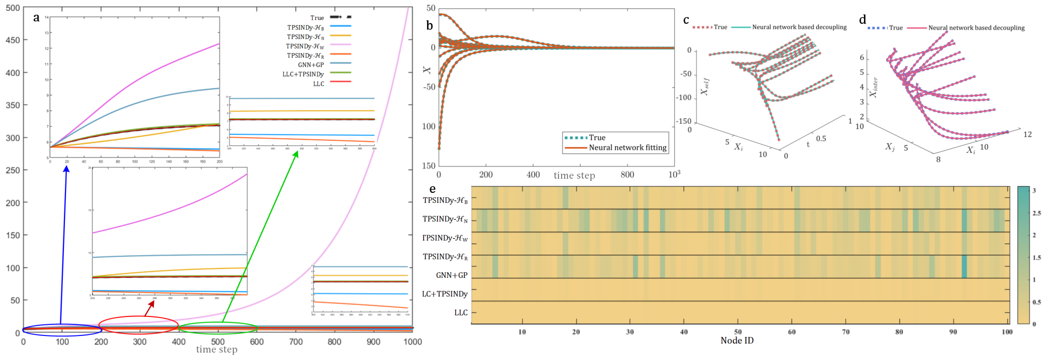


Supplementary Figure 3: Comparison of results produced by various methods on the MI scenario. **a.** Comparison of the difference between the predictive states and the true values using various methods on a node in the MI scenario. **b.** The fitting results of the $\dot{X}_i(t)$ on a node by neural networks. **c.** The decoupling results of the self dynamics on a node ($\hat{Q}_{\theta_1}^{(self)}$). **d.** The decoupling results of the interaction dynamics on a node ($\hat{Q}_{\theta_2}^{(inter)}$). **e.** NED heat map across all nodes.

# V Robustness Evaluation

## A Noisy observations and missing topology

To assess the robustness of our tool, we evaluate its performance under two conditions: noisy observations and missing topological structures. We choose the Kuramoto dynamics [8], a mathematical model for studying how a set of mutually coupled oscillators can synchronize

by interacting with each other, to evaluate the robustness, which can be formulated as

$$\dot{X}_i(t) = \omega_i + \epsilon \sum_{j=1}^{N} A_{i,j} sin(X_j(t) - X_i(t)),$$

where $X_i(t)$ denotes the phase of the $i$-th oscillator, $\epsilon = 0.015$ is the coupling strength, and $\omega_i$ is the natural frequency distributed according to a given normal distribution $\mathcal{N}(1, \sigma)$ with $\sigma = 1$. The time step was set to $\delta t = 0.01$, $T = 5$ and $T_{end} = 100$. We assume that the underlying topological structure is a BA network with $N = 100$.

Complex network systems typically have two sources of noise: measurement and topological noise. The former is mainly arises from inaccurate measurement results from unstable sensors. The latter is caused by the deletion or addition of nodes or edges, resulting in incomplete captured topology [15]. To better understand how the framework performs under different types of noise, we have conducted experiments in Kuramoto dynamic. The amount of noise can be measured by the signal-to-noise ratio (SNR) [16], and the larger its value, the less noise is added. The specific experimental Settings are as follows:

- **Gaussian Noise:** The noise is uniformly superimposed at all time points and exhibits continuous and symmetric random fluctuations. The noise is uniformly superimposed at all time points and exhibits continuous and symmetric random fluctuations.

$$X_i^{\text{noise}}(t) = X_i(t) + \epsilon_{\text{gaussian}}, \epsilon_{\text{gaussian}} \sim \mathcal{N}\left(0, \sigma^2\right), \sigma^2 = \frac{\operatorname{Var}\left(X_i(t)\right)}{10^{SNR/10}}$$

- **Poisson noise:** The noise appears as a discrete disturbance and is more significant in low intensity signals. If the original signal itself is a count, Poisson noise will directly reflect statistical fluctuations.

$$X_i^{\text{noise}}(t) = X_i(t) + \epsilon_{\text{possion}}, \ \epsilon_{\text{possion}} = \text{Possion}(\lambda) - \lambda, \ \lambda = \max\left(\frac{\operatorname{Var}\left(X_i(t)\right)}{10^{\text{SNR}/10}}, 0\right)$$

- **Phase noise:** Noise causes random shifts in phase values, but a phase jump such as a jump from $2\pi$ to $0$ . May introduce discontinuities due to periodic boundary conditions.

$$X_i^{\text{noise}}(t) = \left(X_i(t) + \epsilon_{\text{phase}}\right) \bmod 2\pi, \ \epsilon_{\text{phase}} \sim \mathcal{N}\left(0, \sigma^2\right), \ \sigma^2 = \frac{\operatorname{Var}\left(X_i(t)\right)}{10^{SNR/10}}$$

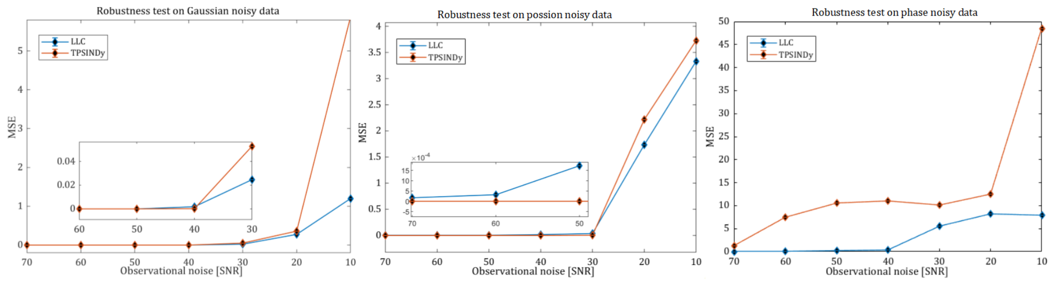


Supplementary Figure 4: The robustness of different noise types in Kuramoto model. The horizontal axis represents the amount of noise which can be measured by the signal-to-noise ratio (SNR), and the larger its value, the less noise is added.

From Supplementary Figure 4, we can see that our LLC is superior to TPSINDy in noise tolerance, regardless of the noise type. In addition, our LLC shows strong robustness in the face of Gaussian noise, indicating that it can effectively "smooth" the observed data, minimize the variance introduced by the noise, and maintain a relatively accurate dynamics fit. For non-Gaussian noise, our LLC still produces stable results with lower levels of noise.

However, the performance of both methods declines compared to their performance with Gaussian noise when higher levels of noise (SNR=30 for possion noise and SNR=40 for phase noise) are introduced, especially in the case of phase noise. This results suggest that the framework may need further optimization when dealing with non-Gaussian noise, which may be left for future work.

To construct incomplete topologies we randomly add or delete a proportion of entries in the true adjacency matrix $A_{i,j}$. The specific noise addition process is as follows:

$$\dot{X}_i(t) = Q^{self}(X_i(t)) + \sum_{j=1}^{N} A'_{i,j} Q^{inter}(X_i, X_j),$$

where $A'_{i,j} = A_{i,j} \cdot (1 - \mathbb{I}\{A_{i,j} = 1 \text{ and } R < \eta\}) + (1 - A_{i,j}) \cdot \mathbb{I}\{A_{i,j} = 0 \text{ and } R < \eta\}$. A random variable $R$ is drawn from a uniform distribution within the range $[0, 1]$. The spurious links probability is designated as $\eta$. $\mathbb{I}$ is an indicator function that assumes the value of 1 when a condition is met and 0 otherwise.

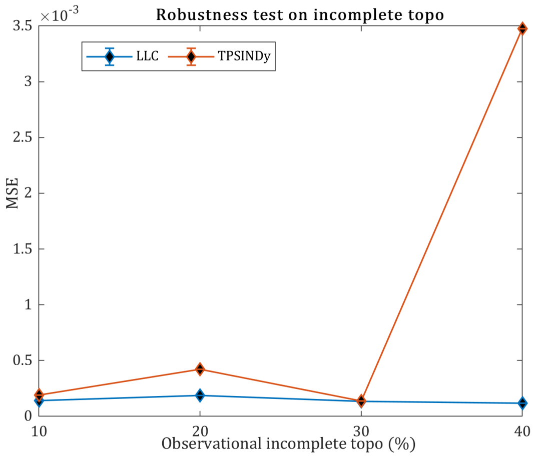


Supplementary Figure 5: Comparison of performance (MSE) as the percentage of spurious topology increases.

Supplementary Figure 5 shows the comparison of performance (MSE) as the percentage of missing topology increases, where the horizontal axis represents the percentage of missing edges. The results indicate that both methods have the extension completion ability, but the performance of our tool can accommodate more missing information.

## B Comparison of different sampling frequencies

To evaluate the impact of different sampling frequencies on the performance, we have conducted experiments on two scenarios, i.e., heat diffusion and Kuramoto. The sampling interval influences the sampling frequency directly. A larger interval results in a lower sampling frequency, leading to sparser data. The specific sampling time interval is from 0.0001, 0.001, 0.01, 0.1 to explore the performance of LLC under different sampling frequencies.

From Supplementary Figure 6, we can see that varying sampling intervals can indeed affect the experimental results. If the sampling interval is too large (e.g. 0.1), the results tend to deteriorate noticeably. Furthermore, different scenarios impact the results in distinct ways, as evidenced by the comparison between the orange and blue lines in Supplementary Figure 6. As mentioned in the main text, we actually utilized the simulated annealing method to optimize for various scenarios, ensuring the reliability and effectiveness of our framework. Empirically, most of the experimental scenarios in the main text choose 0.01 as the time interval to obtain satisfactory performance.

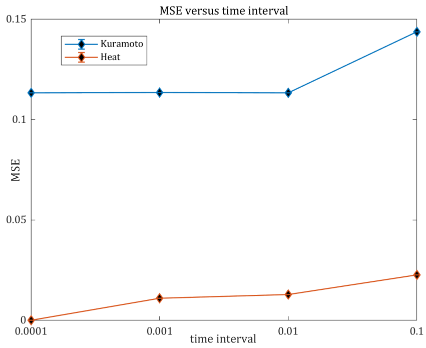


Supplementary Figure 6: Performance comparison of different sampling time intervals in the Heat and Kuramoto scenarios.

## C Comparison of difference methods

We have added a new experiment to compare and analyze the performance of three finite difference methods. The specific Settings are as follows:

- Second order central difference method: The calculation is simple, but the accuracy is low. And it has a relatively low requirement for the smoothness of the function and is suitable for most smooth functions.

$$\dot{X}_i(t) = \frac{X_i(t+\delta_t) - X_i(t-\delta_t)}{2\delta_t}$$

- Fourth order central difference method (5-point approximate difference): The accuracy is significantly higher than that of the second-order method and it is more sensitive to high-frequency noise. It is required that the function be differentiable within a larger neighborhood.

$$\dot{X}_i(t) = \frac{X_i(t-2\delta_t) - 8X_i(t-\delta_t) + 8X_i(t+\delta_t) - X_i(t+2\delta_t)}{12\delta_t}$$

- Sixth order central difference method: It features ultra-high precision and is suitable for extremely smooth functions, but there may be slight noise in the method data.

$$\dot{X}_i(t) = \frac{-X_i(t-3\delta_t) + 9X_i(t-2\delta_t) - 45X_i(t-\delta_t) + 45X_i(t+\delta_t) + 9X_i(t+2\delta_t) - X_i(t+3\delta_t)}{60\delta_t}$$

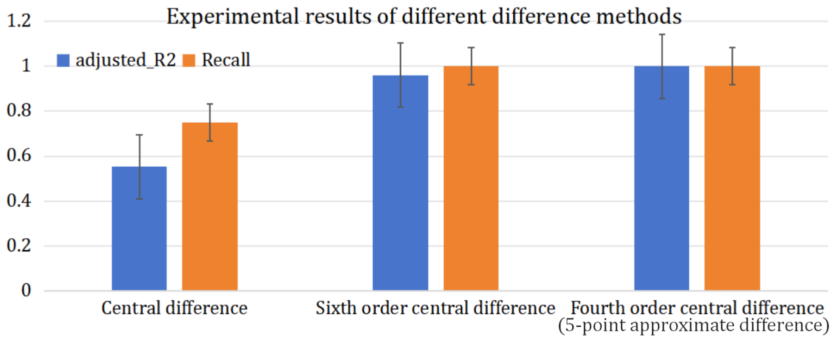


Supplementary Figure 7: Comparison of the average performance of different finite difference methods in six one-dimensional network dynamic scenarios.

Supplementary Figure 7 shows the average results using these three difference methods over six network dynamics scenarios under the same experimental conditions. The results indicate that the framework performs well with medium accuracy when using the fourth-order difference approximation. However, while the sixth-order central difference method can effectively reconstruct the skeleton of equation, its prediction accuracy is lower than that of the LLC method. This discrepancy arises because the LLC requires the recovered equation to be sufficiently smooth and the boundary conditions to be extendable. Additionally, the second-order central method exhibits a large error due to its lower accuracy. Therefore, this work empirically selects the fourth-order difference approximation (a five-point approximate difference method) as the derivative calculation method to strike a balance between accuracy and efficiency.

# VI An Ablation Experiment to Evaluate the Effectiveness of the Signal Decoupling Architecture Design

In order to verify the necessity of combination of $\psi^{g0}(X_i, X_j)$ and $\psi^{g1}(X_i)\psi^{g2}(X_j)$ in the neural network in the first part of LLC. We have constructed an ablation experiment to verify the necessity. The specific settings are as follows: we compare the effects of the five variant methods on network dynamics scenarios, including: Biochemical (Bio), Gene regulatory (Gene), Mutualistic Interaction (MI), Neural (Neur), and Epidemic (Epi) dynamics.

- $g_0$ **moudle:** It only keeps the $\psi^{g_0}$ to process the input signal for capturing interactive dynamics.
- $g_0^{deep}$ **moudle:** The same architecture as the $\psi^{g_0}(X_i, X_j)$ was used, but the number of hidden layers was set 5 to verify whether the neural network with higher depth could improve the expression effect of interactive information.
- $g_1 * g_2$ **moudle:** It uses only the $\psi^{g_1}(X_i)\psi^{g_2}(X_j)$ part of the original design to capture interactive dynamics.
- $g_1^{deep} * g_2$ **moudle:** The $\psi^{g_2}(X_j)$ part is kept unchanged on the original $\psi^{g_1}(X_i)\psi^{g_2}(X_j)$ structure, and the number of hidden layers of $\psi^{g_1}(X_i)$ is set 5 to verify the role of $\psi^{g_1}(X_i)$ depth in capturing complex dynamics.
- $g_1 * g_2^{deep}$ **moudle:** The $\psi^{g_1}(X_j)$ part is kept unchanged on the original $\psi^{g_1}(X_i)\psi^{g_2}(X_j)$ structure, and the number of hidden layers of $\psi^{g_2}(X_j)$ is set 5 to verify the role of $\psi^{g_2}(X_j)$ depth in capturing complex dynamics.

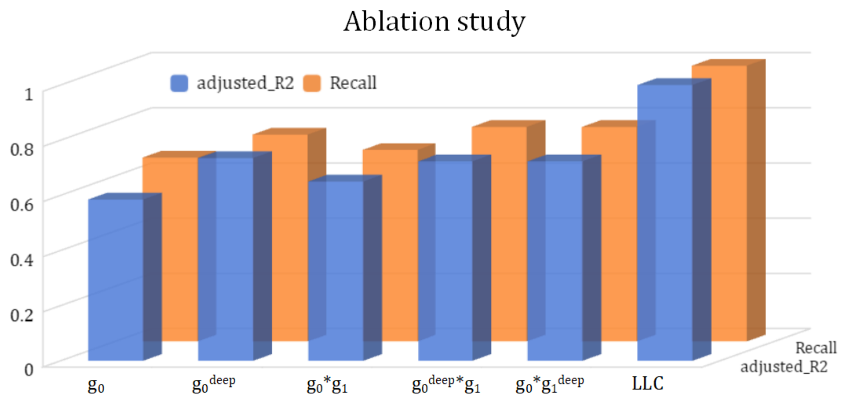


Supplementary Figure 8: Comparison results of average performance across six network dynamics scenarios from five variants and our LLC.

Supplementary Figure 8 shows the average performance of five variants and our LLC in terms of adjusted $R^2$ and Recall. We see that the variants with independent use of

$\psi^{g_0}(X_i, X_j)$ and $\psi^{g_1}(X_i)\psi^{g_2}(X_j)$ perform poorly, and the independent increase of depth can indeed enhance the performance, but the improvements are still limited. Our LLC considers both multiplicative decomposable and non-decomposable cases by combining $\psi^{g_0}(X_i, X_j)$ and $\psi^{g_1}(X_i)\psi^{g_2}(X_j)$, which may make model learning easier and obtain the optimal performance empirically.

# VII Inferring Higher-Order Network Dynamics with Multi-Node Interaction

To verify the effectiveness of the proposed method on higher-order dynamics, LLC is applied to the continuous dynamic [17] development of consensus processes in higher-order networks, where the node dynamics are described by the following system of equations:

$$\dot{X}_i(t) = \sum_{jk} A_{i,j,k} \exp\left(l|X_k - X_j|\right)\left[(X_j - X_i) + (X_k - X_i)\right]$$

$X_i(t)$ is the state of node $i$ at time $t$ (such as opinion, preference, etc.), and $A_{ijk} \in (0,1)^{N\times N\times N}$ is a 3-hypergraph adjacency tensor representing triadic interactions. $\exp\left(l|X_k - X_j|\right)$ is a nonlinear scaling function that modulates the effects of $j$ and $k$ on node $i$. $l$ is a parameter that controls the sensitivity of opinion differences: If $l > 0$, the node prefers to synchronize with neighbors with similar opinions (*homophily*). If $l < 0$, the node may be influenced by neighbors with large opinion differences (*heterophily*). $(X_j - X_i) + (X_k - X_i)$ indicates that the state of node $i$ is jointly influenced by $j$ and $k$, similar to the action of the higher-order Laplacian. Under the ER network topology complement setting, we generate random trajectories for $N = 100$, $T = 0.03$ and $\delta t = 0.0001$.The dynamical interaction equation inferred by our approach from the data generated on a ER network with $l = 1$ is

$$\dot{X}_i(t) = \sum_{jk} A_{i,j,k} \exp\left(|X_k - X_j|\right)\left[(X_j - X_i) + (X_k - X_i)\right]$$

Another case for higher-order interaction dynamics is opinion dynamics [18]. The system is tested on scenarios containing self-dynamics and higher-order interactions. This scenario describes that in a social network, individual opinion evolution is affected by a three-person group, and the specific equation is as follows:

$$\dot{X}_i(t) = -\mu X_i + \sum_{jk} A_{i,j,k} X_j X_k (1 - |X_i|)$$

where $X_i \in [-1, 1]$ represents the opinion tendency of an individual $i$, and is a third-order tensor (*3-hypergraph adjacency tensor*), denoted as $A_{ijk} \in (0,1)^{N\times N\times N}$, which represents the influence weight of a trio. $\mu$ denotes the individual opinion decay rate, and $\gamma$ denotes the group coupling strength. When the opinion of individual $i$ is consistent with the group opinion ($X_j X_k > 0$), its opinion is shifted towards the group direction. The nonlinear term $(1 - |X_i|)$ prevents the opinion from exceeding the boundary $[-1, 1]$. We set the parameters as follows: $\mu = 0.3, \gamma = 1, N = 100$, and the network topology is a random network. The equations inferred by our approach from the data generated on ER network is

$$\dot{X}_i(t) = -0.298 X_i + \sum_{jk} A_{i,j,k} X_j X_k (1.006 - |X_i|)$$

Supplementary Figure 9 shows the results under two scenarios in terms of various evaluation indicators. By properly extending the signal decoupling module to support multi-nodes interactions, we can accurately infer higher-order components using our LLC. This demonstrates its flexibility, as our LLC can effectively handle high-order network dynamics involving multi-nodes.

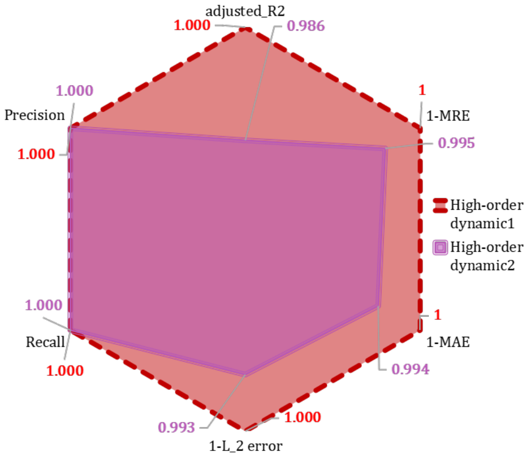


Supplementary Figure 9: Performance evaluation of two high-order dynamics scenarios under six evaluation indicators.

# VIII Multi-Dimensional and Heterogeneous Network Dynamics

## A FitzHugh-Nagumo dynamics

The FitzHugh-Nagumo model (FHN) [19] is a neuron model that describes the excitatory behavior of neurons. As a simplified version of the Hodgkin-Huxley model, it is primarily used to study neuron action potentials. It can be defined by a set of two ordinary differential equations that capture the main features of excitability in neural membrane dynamics. These equations governing the dynamics are expressed as:

$$\begin{cases} \dot{X}_{i,1}(t) = X_{i,1}(t) - X_{i,1}(t)^3 - X_{i,2}(t) - \epsilon \sum_{j=1}^{N} A_{i,j} \frac{X_{j,1}(t) - X_{i,1}(t)}{K_{in}}, \\ \dot{X}_{i,2}(t) = a + bX_{i,1}(t) + cX_{i,2}(t) \end{cases}$$

where the first component $X_{i,1}$ denotes the membrane potential containing the self and interaction dynamics, $K_{in}$ is the in-degree of neuron $i$ (denoting the number of afferent connections to node $i$), and $\epsilon = 1$. The second component $X_{i,2}$ denotes the recovery variable, where $a = 0.28$, $b = 0.5$, $c = -0.04$. We set the underlying topology to BA network with $N = 100$ and terminal time $T = 3$, $T_{end} = 100$ and time step $\delta t = 0.01$.

For the detailed setting of the TPSINDy in this dynamics, we make the basic function terms in the TPSINDy contain the polynomial terms, e.g., $X_i, X_i^2, X_j, X_j^2, X_iX_j, X_j - X_i$, their associated trigonometric counterparts, e.g., $sin(X_i), sin(X_j), sin(X_iX_j), sin(X_j - X_i), X_i sin(X_j)$, and the rescaling functions, e.g., $\frac{X_i}{K_{in}}, \frac{X_j}{K_{in}}, \frac{X_iX_j}{K_{in}}$, and $\frac{X_j - X_i}{K_{in}}$.

Moreover, we apply our LLC to FHN on a Barabási-Albert network and two empirical networks, including the C. elegans [12] and Drosophila [13], to assess the influence of different topologies on the results. The experimental setup is delineated as follows. During the training phase, we conduct experiments across three network topologies: BA, C.elegans, and Drosophila. These topologies were chosen to represent different types of complex network structures: a scale-free synthetic network (BA) and two real-world biological networks (C. elegans and Drosophila). This diversity allows for a comprehensive evaluation of how our LLC performs under varying structural conditions. The initial values for each node are drawn from a normal distribution $N(0, 1)$, with a time step interval of 0.01 and a total simulation duration of T=3. We systematically identify the governing equations for each network topology and subsequently perform comparative analyses on a selected network structure (e.g., BA) to evaluate the resulting experimental trajectories and node-wise errors. The comprehensive results of these analyses are depicted in Supplementary Figure 10(a). The results clearly demonstrate that our tool effectively identifies the correct equations across both synthetic and real-world network structures. The long-term predictive trajectories

produced by the discovered equations derived from different topologies exhibit remarkable consistency. Supplementary Figure 10(b) illustrates the node error distributions, revealing that approximately 90% of nodes display NED errors near zero, with only a few nodes showing errors around 0.1. The relative errors associated with the inferred equation coefficients are presented in Supplementary Figure 10(c), where maximum coefficient discrepancies do not exceed 3%. Finally, spatial trajectory comparisons depicted in Supplementary Figure 10(d) indicate that LLC demonstrates robust fitting capabilities across all dimensions.

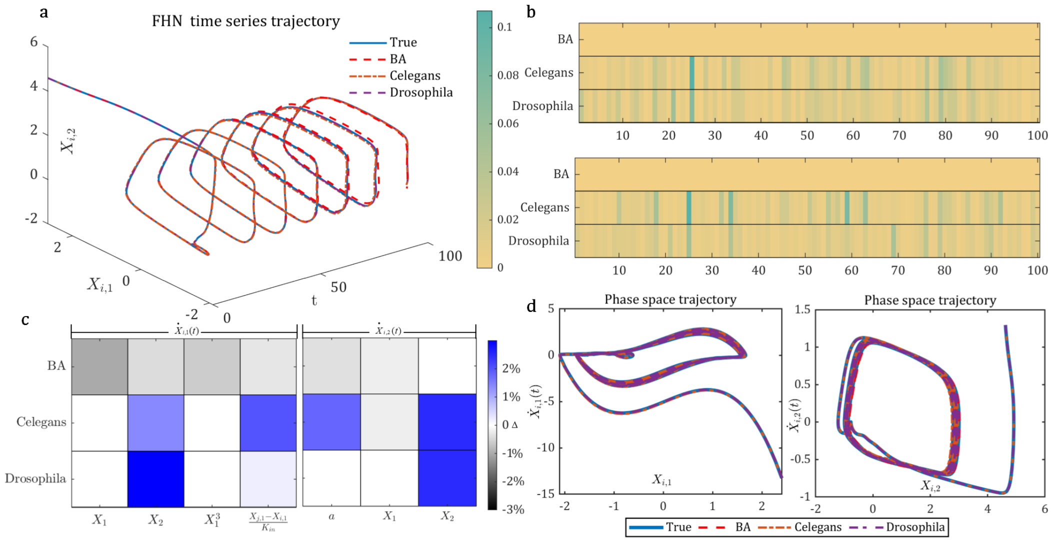


Supplementary Figure 10: Recognition results on different topological structures. **a.** Comparison of node activities for discovered equations. **b.** NED values of nodes. **c.** Comparison of coefficient errors for discovered equations. **d.** Phase space trajectories of a specified node. The results empirically demonstrate that our tool can obtain satisfactory equations and predictive trajectories on both synthetic and empirical topologies.

# B Predator-prey system

The predator-prey (PP) model is a heterogeneous system [9], where the node state represents the position of each individual. Nodes are classified into two roles: a single predator ($i = 0$) and multiple preys ($i > 0$), leading to three types of pairwise interactions: predator-prey, prey-predator, and prey-prey. The interactions between prey are modeled to exhibit paired short-range repulsion and long-range attraction. Specifically, the form of the prey-prey interaction is defined as follows:

$$F_{i,\text{prey-prey}} = \frac{1}{N} \sum_{j=1, j \neq i}^{N} \left( \frac{1}{|X_i(t) - X_j(t)|^2} - a \right) (X_i(t) - X_j(t)).$$

Here, $\frac{X_i(t)-X_j(t)}{|X_i(t)-X_j(t)|^2}$ represents a Newtonian short-range repulsive force directed from $X_i(t)$ to $X_j(t)$, while $-a(X_i(t)-X_j(t))$ corresponds a linear long-range attractive force in the same direction. Although more generalized attraction-repulsion dynamics could be considered, we focus on this specific form due to its ability to yield clearer and more definitive results Assuming the presence of a predator, we denote its position to be denoted by $X_0(t)$. The predator's interaction with the prey is modeled as a repulsive force, expressed as:

$$F_{j,\text{predator-prey}} = b \frac{X_j(t) - X_0(t)}{|X_j(t) - X_0(t)|^2},$$

where $b$ represents the intensity of the repulsive force. Based on the above considerations, the predator-prey system can be formulated as follows:

$$\begin{cases} \dot{X}_0(t) = \frac{c}{N}\sum_{j=1}^{N} \frac{X_j(t)-X_0(t)}{|X_j(t)-X_0(t)|^2}, \\ \dot{X}_i(t) = b\frac{(X_i(t)-X_0(t))}{|X_i(t)-X_0(t)|^2} + \frac{1}{N}\sum_{j=1}^{N}\left(\frac{(X_i(t)-X_j(t))}{|X_i(t)-X_j(t)|^2} + a\left(X_j(t)-X_i(t)\right)\right), i > 0, \end{cases}$$

where we set $a$, $b$, and $c$ to 1.0, 0.2, and 0.7, respectively. For the underlying topology, we

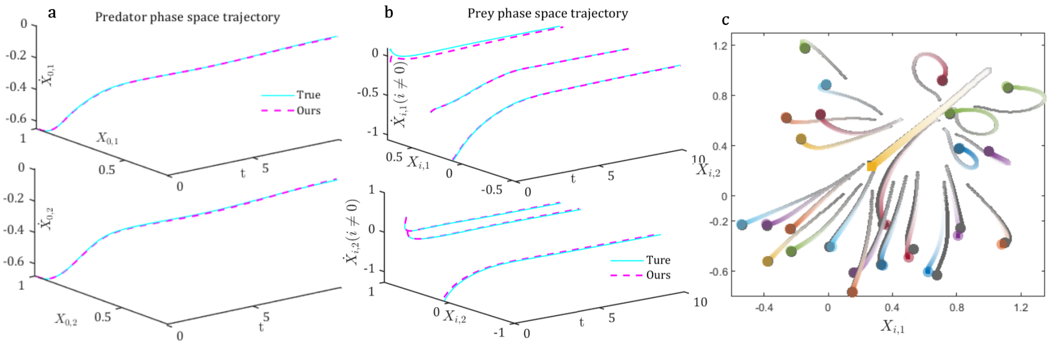


Supplementary Figure 11: Comparison of trajectories of the Predator-Prey system. **a.** Comparison of phase trajectories for each dimension of the predator. **b.** Comparison of phase trajectories for each dimension of a prey. **c.** The comparison of predator and prey swarm trajectories is shown, where the gray trajectories represent those generated by the true equation, and the colored trajectories are generated by the equations discovered by our LLC. Squares represent the predator, and circles represent the prey.

consider a fully connected structure in this system with $N = 101$.

For the compared TPSINDy, we utilizes a comprehensive function library consisting of various terms, including polynomial terms $X_i, X_i^2, X_j, X_j^2, X_iX_j, X_j - X_i$, exponential functions $e^{X_i}, e^{X_j}, e^{X_iX_j}, e^{X_i-X_j}$, fractional functions $\frac{1}{X_i}, \frac{1}{X_j}, \frac{1}{X_iX_j}$, and repulsive force terms $\frac{X_i(t)-X_j(t)}{|X_i(t)-X_j(t)|^2}, \frac{X_i(t)-X_0(t)}{|X_i(t)-X_0(t)|^2}$.

Supplementary Figure 11 shows the comparison of trajectories of the predator-prey system. From Supplementary Figure 11(a, b), it is evident that the proposed LLC fits the real spatial trajectories very well. Additionally, we demonstrate how well the inferred equations fit the predicted and actual trajectories. Supplementary Figure 11(c) provides an example with 50 agents, where the colored portion represents the predicted trajectories and the gray portion represents the actual trajectories. Both trajectories coincide to a large extent, indicating that the equation discovered by LLC provides an accurate prediction for this system.

# IX Chaotic Networks Dynamics

## A Lorenz system

The coupled Lorenz system [20] exhibits chaotic behavior, particularly sensitive dependence on initial conditions, meaning that even small changes in the initial conditions can result in drastically different system dynamics To verify whether the proposed LLC is sensitive to initial values, we apply it to the coupled Lorenz system system with different initial conditions and examine whether the discovered governing equations are consistent.

Specifically, we apply our LLC to a coupled Lorenz system [20] governed by

$$\begin{cases} \dot{X}_{i,1}(t) = a(X_{i,2}(t) - X_{i,1}(t)) + \epsilon\sum_{j=1}^{N} A_{i,j}(X_{j,1}(t) - X_{i,1}(t)), \\ \dot{X}_{i,2}(t) = rX_{i,1}(t) - X_{i,1}(t)X_{i,3}(t) - X_{i,2}(t), \\ \dot{X}_{i,3}(t) = X_{i,2}(t)X_{i,1}(t) - bX_{i,3}(t), \end{cases}$$

where $a = 10$, $\epsilon = 0.05$, $r = 28$ and $b = \frac{10}{3}$ are system parameters. Here, the interactions are assumed to occur between the first component $X_{i,1}$ without lack of generality. Then, we employ a BA network with $N = 100$ as the topological structure to generate the dynamics data of the Lorenz. We set the end time $T = 3$, $T_{end} = 100$ and time step $\delta t = 0.01$.

We establish three distinct initial conditions as follows:

- Initial condition 1: A Gaussian distribution with zero mean and one variance;
- Initial condition 2: A uniform distribution within the range (0, 2);
- Initial condition 3: A fixed initial value of 0.1.

In addition to the consistent equations discovered at three different initial values presented in the main text, we also demonstrate the inferring results of a coupled Lorenz system with 2,500 attractors under different initial conditions, as shown in Supplementary Figure 12. It is evident that despite only minor variations in the initial conditions, there are pronounced differences in the final states, as demonstrated by comparing the first two rows. Notably, our LLC yields results similar those presented in the second row regardless of varying initial conditions, as shown by comparing the second and third rows. To better illustrate the differences between the ground truth and predicted outcomes, we visualize the error at the final state, demonstrating that our LLC can make predictions with an error margin approaching zero.

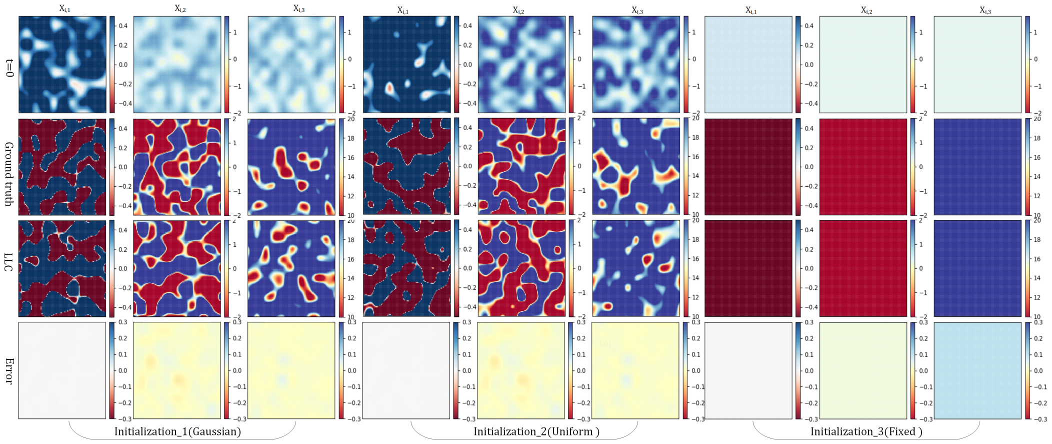


Supplementary Figure 12: Inferring results of a coupled Lorenz system with 2,500 attractors under different initial conditions. We rearranged all attractors in a $50 \times 50$ grid format. The first row shows the initial state of each dimension, i.e., $t = 0$. The second row shows the final state ($T_{end} = 30$) of the ground truth equation under different initial conditions. The third row shows the predictive final state ($T_{end} = 30$) produced by equations discovered by the LLC under different initial conditions. The last row shows the error between the predictive final state and the ground truth.

## B Rössler system

We extend our study to a coupled Rössler system, a classical model frequently employed to explore chaotic dynamics and synchronization in complex networks. The data for chaotic states is generated using the governing equations defined as

$$\begin{cases} \dot{X}_{i,1}(t) = -X_{i,2} - X_{i,3} + \epsilon \sum_{j=1}^{N} A_{i,j}(X_{j,1} - X_{i,1}) \\ \dot{X}_{i,2}(t) = X_{i,1} + aX_{i,2} \\ \dot{X}_{i,3}(t) = b + X_{i,3}(X_{i,1} - c) \end{cases} \tag{I1}$$

where $\epsilon = 0.15$, $a = 0.2$, $b = 0.2$ and $c = 5.7$ are system parameters. We employ a BA network with $N = 100$ as the topological structure to produce the dynamics data of the system. We set the end time $T = 3$, $T_{end} = 100$ and time step $\delta t = 0.01$.

We set up the function library for the comparison algorithm TPSINDy, which includes the following: the polynomial terms, e.g., $X_i$, $X_i^2$, $X_j$, $X_j^2$, $X_iX_j$, $X_j - X_i$, exponential functions, e.g., $e^{X_i}, e^{X_j}, e^{X_iX_j}, e^{X_i - X_j}, X_i e^{X_j}$, trigonometric terms, e.g., $sin(X_i), sin(X_j), sin(X_iX_j), sin(X_j - X_i), X_i sin(X_j)$, and activation functions, e.g., $sigmod(X_i), sigmod(X_j), sigmod(X_iX_j), sigmod(X_j - X_i), X_i sigmod(X_j)$.

Supplementary Figure 13 shows the comparison of inferred results on a coupled Rössler system. LLC and LLC+TPSINDy closely fit the original trajectories, while TPSINDy only maintains a good fit during the initial segment, differing significantly from the true state after approximately 3,000 steps, as shown in Supplementary Figure 13(a).

The comparison of coefficients of the equations discovered by different methods is shown in Supplementary Figure 13(b). We find that the coefficients of the equation discovered by TPSINDy slightly differ from the true coefficients. This also indicates that, for chaotic systems, even a small change in the coefficients can have a significant impact on predictive states, particularly in long-term forecasting. Supplementary Figure 13(c) shows the NED values of each node under different methods, demonstrating that our LLC achieves relatively high accuracy. For the first two dimensions, TPSINDy consistently yields NED values greater than 0.1, whereas the NED values produced by our LLC predominantly remain below 0.06. In the third dimension, the proposed LLC performs worse than the TPSINDy method. However, the combination of LLC and TPSINDy effectively mitigates this limitation, yielding NED values close to 0.

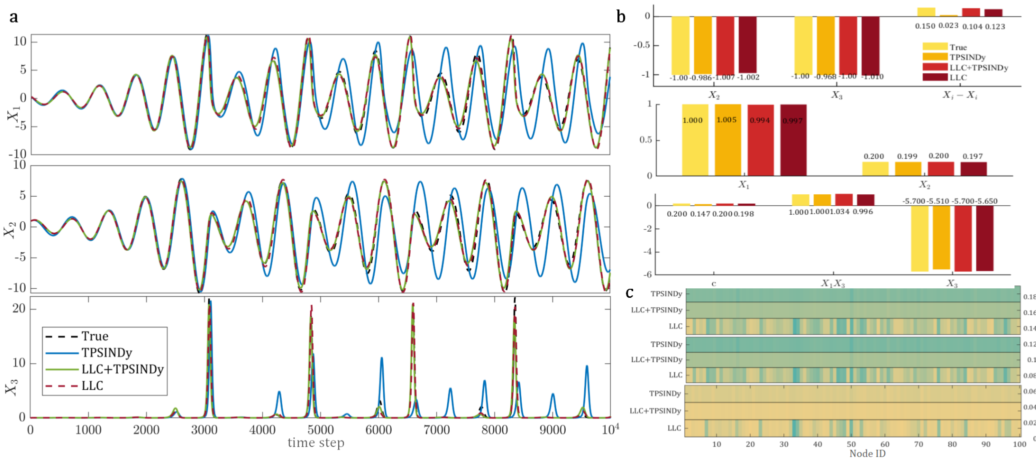


Supplementary Figure 13: Comparison of inferred results on a coupled Rössler system. **a.** Comparison of predictive states from different methods in each dimension changes over time. **b.** Comparison of coefficients of the equations discovered by different methods. **c.** NED values of each node under different methods.

Compared to the TPSINDy on the Rössler system, our LLC reconstructs more accurate governing equations (Fig. 5(c)) and achieves smaller predictive errors (Fig. 5(d)). Analysis of the state transition behavior of the Rössler system shows that the inferred and true equations' bifurcation diagrams closely align. This consistency in period-doubling patterns and bifurcation points highlights the transition sequence from period-1 to period-2, to period-4, to chaos, followed by period-3, and ultimately back to chaos (see Fig. 5(e)).

To further intuitively demonstrate the chaotic nature of the system, we utilize a Poincaré surface to construct a bifurcation diagram, as shown in Fig. 5(e) in the main text. Specifically, we select $X_1 = 0.1$ as the section, recording intersection points as the trajectory crosses this plane. By documenting a substantial number of intersection points, we can generate the Poincaré section. If the system exhibits periodic motion, a finite number of points will appear on the Poincaré section. For instance, period-2 will manifest as two points on the section. Conversely, if the system exhibits chaotic motion, the Poincaré section will

display a large number of densely packed points, revealing a complex structure. Supplementary Figure 14 visualizes the period-doubling and chaos of the Rössler system, showing the consistent limit cycles and chaotic phenomena.

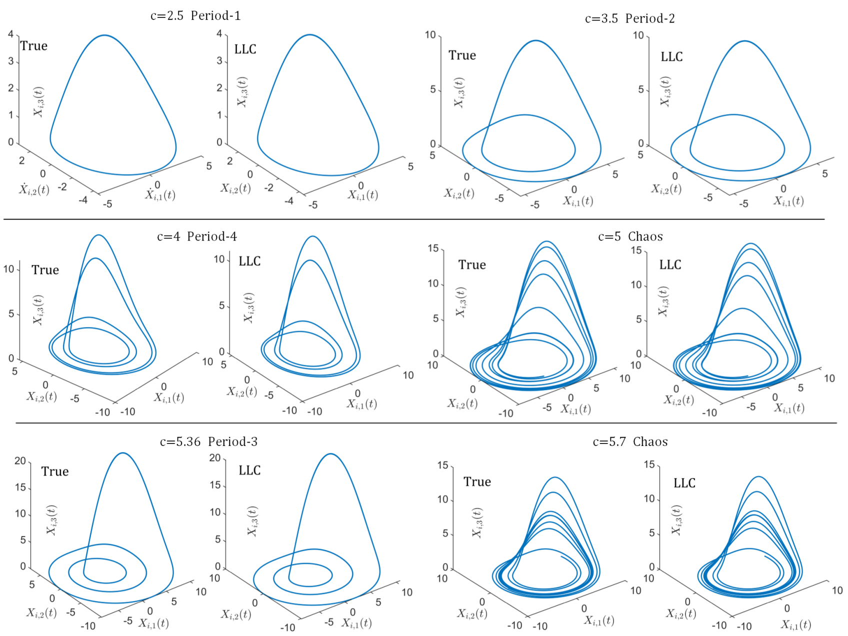


Supplementary Figure 14: Period-doubling and chaos of the Rössler system. Comparison of limit cycles at period-1 with $c = 2.5$, period-2 with $c = 3.5$, period-4 with $c = 4$, chaos with $c = 5$, period-3 with $c = 5.36$, and chaos again with $c = 5.7$.

# X Empirical Systems

For the real-world systems, we use two datasets from different domains, including the daily global propagation data of real diseases [21] and real-world crowd trajectory dataset [22].

## A Real-world global epidemic transmission

We collect daily global spreading data on COVID-19 [21], and use the worldwide airline network retrieved from OpenFights [23] as a directed and weighted empirical topology to build an empirical system of real-world global epidemic transmission. Only early data before government intervention, i.e., the first 45 days, are considered here to maintain the spread characteristics of the disease itself. For example, if a country reports its first case on January 19, data from January 19 to March 3 are used. It is worth noting that although the start of transmission varies across countries, in the interaction dynamics $Q_{i,j}^{(inter)}$, time $t$ corresponds to the same calendar date for all nodes. Our setup is consistent with [24], including the selection of the function library for the TPSINDy. Supplementary Table 8 lists the governing equations found by different methods. The performance of LLC+TPSINDy and LLC$_{total}$ is similar and clearly better than that of TPSINDy. This indicates that our newly discovered disease transmission equations are more suitable for describing the epidemic spread. It also aligns with the intuition that while the functional form of each region is the same, the parameters may differ. Supplementary Figure 15 shows the comparative results of the number of cases over time in sufficient countries or regions generated by TPSINDy, LLC$_{each}$, LLC$_{total}$ and LLC$_{each}$+TPSINDy, demonstrating the effectiveness of our LLC-based in discovering new symbolic models for real scenario with unknown dynamics.

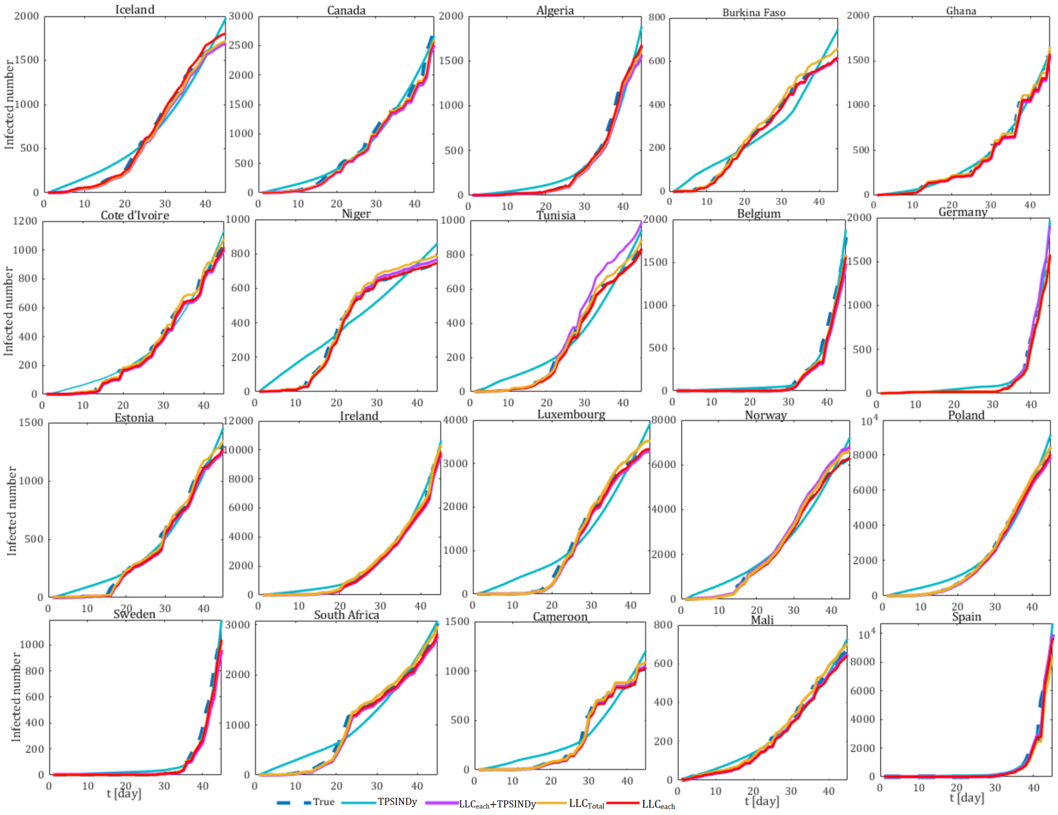


Supplementary Figure 15: Comparative results of the number of cases over time in sufficient countries or regions generated by TPSINDy, LLC$_{each}$, LLC$_{total}$, and LLC$_{each}$+TPSINDy.

Supplementary Table 8: Comparison of governing equations found by different methods in real-world epidemic system

| Country | TPSINDy | LLC$_{each}$+TPSINDy | LLC$_{total}$ | LLC$_{each}$ |
|---|---|---|---|---|
| Templates | $\dot{X}_i(t) = aX_i + \sum_{j=1}^{N} A_{ij} \frac{b}{e^{-(X_j - X_i)}}$ | $\dot{X}_i(t) = aX_i + \sum_{j=1}^{N} A_{ij} \frac{b + c_0 X_i}{c_1 X_i + c_2 X_j + c_3}$ | $\dot{X}_i(t) = aX_i + \sum_{j=1}^{N} A_{ij} \frac{bX_j}{e^{-cX_j/X_i}}$ | N/A |
| Iceland | $a = 0.042$, $b = 708.343$ | $a = 1, b = -0.224$ $c_0 = 2.600, c_1 = 2.277$ $c_2 = 2.894, c_3 = 5.444$ | $a = 1.015$, $b = 0.021$ $c = -15.682$ | $\dot{X}(t) = 1.060X_i + 2.512e^{-(11.999X_i/X_j)} + \sum_{j=1}^{N} A_{ij} \frac{exp{-}X_j/(8.620X_i + 24.685)}{X_j/(1.886X_i - X_i + 1.689)}$ |
| Canada | $a = 0.080$, $b = 28.534$ | $a = 0.980, b = 66.174$ $c_0 = 9.385, c_1 = 3.157$ $c_2 = 4.400, c_3 = 3.955$ | $a = 1.015$, $b = 0.018$ $c = -27.418$ | $\dot{X}(t) = 1.070X_i + \sum_{j=1}^{N} A_{ij} (0.342X_i - 2.268) \frac{e^{-24.733/X_j}}{X_i}$ |
| Algeria | $a = 0.112$, $b = 123.971$ | $a = 1, b = 2.530$ $c_0 = 3.551, c_1 = 1.130$ $c_2 = 4.534, c_3 = 5.846$ | $a = 1.037$, $b = 0.018$, $c = -16.059$ | $\dot{X}(t) = 1.059X_i + \sum_{j=1}^{N} A_{ij} 0.02\, exp(\frac{24.519 - 5.011X_i}{X_j})$ |
| Burkina Faso | $a = 0.011$, $b = 922.518$ | $a = 1.049, b = 4.028$ $c_0 = 4.150, c_1 = 1.261$ $c_2 = 4.514, c_3 = 5.989$ | $a = 1.014$, $b = 0.068$, $c = -27.418$ | $\dot{X}(t) = 1.057X_i + \sum_{j=1}^{N} A_{ij} \frac{e^{8.625X_j - 4755.94}}{X_i}$ |

continue 8

| **Country** | **TPSINDy** | **LLC$_{each}$+TPSINDy** | **LLC$_{total}$** | **LLC$_{each}$** |
|---|---|---|---|---|
| Ghana | $a = 0.076,$ $b = 282.872$ | $a = 1.079, b = 2.525$ $c_0 = 3.762, c_1 = 1.137$ $c_2 = 4.536, c_3 = 5.966$ | $a = 1.019,$ $b = 0.231,$ $c = -30.440$ | $\dot{X}(t) = 1.056X_i + \sum_{j=1}^{N} A_{ij} \frac{X_j(0.366X_i - 2.771)}{X_j^2 + 2370.96}$ |
| Cote d'Ivoire | $a = 0.067,$ $b = 189.751$ | $a = 0.949, b = 5.641$ $c_0 = 2.223, c_1 = 4.119$ $c_2 = 2.905, c_3 = 3.377$ | $a = 1.014,$ $b = 0.676,$ $c = -27.418$ | $\dot{X}(t) = 1.066X_i + 7.99 - \frac{7.99}{X_i} + \sum_{j=1}^{N} A_{ij} \frac{0.995X_j - 2.771}{X_j - 1.031X_i + 3095.15}$ |
| Niger | $a = 0.028,$ $b = 970.443$ | $a = 1.042, b = -4.020$ $c_0 = 1.425, c_1 = 1.136$ $c_2 = 4.539, c_3 = 6.013$ | $a = 1.015,$ $b = 0.676$ $c = -27.418$ | $\dot{X}(t) = 1.056X_i + \sum_{j=1}^{N} A_{ij} 0.004(X_j - X_i)$ |
| Tunisia | $a = 0.049,$ $b = 77.143$ | $a = 1.202, b = 29.153$ $c_0 = 29.292, c_1 = 4.914$ $c_2 = 3.667, c_3 = 3.851$ | $a = 1.014,$ $b = 0.068$ $c = -27.419$ | $\dot{X}(t) = 1.059X_i + 1 + \sum_{j=1}^{N} A_{ij} 0.470e^{-32.989/X_j}$ |
| Belgium | $a = 0.237,$ $b = 8.970$ | $a = 0.997, b = 17.603$ $c_0 = 5.261, c_1 = 3.195$ $c_2 = 4.579, c_3 = 3.974$ | $a = 1.051,$ $b = 0.068$ $c = -27.419$ | $\dot{X}(t) = \frac{X_i(0.051X_i + 6.17)}{0.049X_i + 4.909} + \sum_{j=1}^{N} A_{ij} \frac{0.356X_i}{X_j + 102}$ |
| Germany | $a = 0.291,$ $b = -8.556$ | $a = 1.289, b = 0$ $c_0 = 2.509, c_1 = 3.407$ $c_2 = 4.261, c_3 = 4.413$ | $a = 0.985,$ $b = 0.676$ $c = -27.419$ | $\dot{X}(t) = 1.068X_i + \sum_{j=1}^{N} A_{ij} \frac{X_i - 0.071X_j - 2.458}{2.142X_j + 74.374}$ |
| Estonia | $a = 0.058,$ $b = 358.731$ | $a = 1, b = 14.153$ $c_0 = 4.249, c_1 = 2.093$ $c_2 = 2.591, c_3 = 5.362$ | $a = 1.014,$ $b = 1.068$ $c = -27.419$ | $\dot{X}(t) = 1.057X_i + \sum_{j=1}^{N} A_{ij} \frac{2.298X_j - 9.732}{2.142X_j + 68.991}$ |
| Ireland | $a = 0.099,$ $b = 153.027$ | $a = 1.031, b = 9.625$ $c_0 = -4.025, c_1 = 1.144$ $c_2 = 4.566, c_3 = 5.992$ | $a = 1.017,$ $b = 1.067$ $c = -27.419$ | $\dot{X}(t) = 1.070X_i + \sum_{j=1}^{N} A_{ij} \frac{(X_j - 5.211)(X_j - 1.327)}{0.142X_j(X_j - 1.327) + 5.211}$ |
| Luxembourg | $a = 0.047,$ $b = 623.419$ | $a = 1.093, b = 1.193$ $c_0 = 2.382, c_1 = 0.884$ $c_2 = 4.275, c_3 = 5.948$ | $a = 1.017,$ $b = 0.067$ $c = -27.419$ | $\dot{X}(t) = 1.074X_i + \sum_{j=1}^{N} A_{ij} 0.006X_j$ |
| Norway | $a = 0.042,$ $b = 515.3854$ | $a = 1.092, b = 55.859$ $c_0 = 223.949, c_1 = 2.250$ $c_2 = 3.977, c_3 = 4.234$ | $a = 1.019,$ $b = 0.067$ $c = -27.419$ | $\dot{X}(t) = 1.071X_i + \sum_{j=1}^{N} A_{ij} 0.001X_j$ |
| Poland | $a = 0.072,$ $b = 201.713$ | $a = 0.972, b = 10.025$ $c_0 = 9.100, c_1 = 3.183$ $c_2 = 4.401, c_3 = 3.998$ | $a = 1.018,$ $b = 0.068$ $c = -27.419$ | $\dot{X}(t) = 1.068X_i + \sum_{j=1}^{N} A_{ij} 0.00$ |
| Sweden | $a = 0.276,$ $b = 7.410$ | $a = 1.008, b = 5.169$ $c_0 = 3.654, c_1 = 1.168$ $c_2 = 4.567, c_3 = 5.985$ | $a = 1.085,$ $b = 0.067$ $c = -27.419$ | $\dot{X}(t) = 1.064X_i + \sum_{j=1}^{N} A_{ij} \frac{0.354 + e^{-(0.354X_j + 15.327)}}{X_i}$ |

continue 8

| **Country** | **TPSINDy** | **LLC$_{each}$+TPSINDy** | **LLC$_{total}$** | **LLC$_{each}$** |
|---|---|---|---|---|
| South Africa | $a = 0.045$, $b = 652.840$ | $a = 0.993, b = 0.129$ $c_0 = 0.609, c_1 = 3.526$ $c_2 = 4.743, c_3 = 3.975$ | $a = 1.017$, $b = 0.067$ $c = -27.419$ | $\dot{X}(t) = 1.073X_i + \sum_{j=1}^{N} A_{ij} \left(\frac{0.304(X_i - 1)}{X_j} - \frac{0.023}{X_i^2}\right)$ |
| Cameroon | $a = 0.064$, $b = 417.863$ | $a = 1.030, b = 2.193$ $c_0 = 2.930, c_1 = 1.368$ $c_2 = 4.530, c_3 = 6.010$ | $a = 1.012$, $b = 0.067$ $c = -27.419$ | $\dot{X}(t) = 1.057X_i + \sum_{j=1}^{N} A_{ij} 0.470e^{-26.485/X_j}$ |
| Mali | $a = 0.052$, $b = 201.836$ | $a = 0.997, b = 11.505$ $c_0 = 5.874, c_1 = 3.008$ $c_2 = 4.366, c_3 = 3.936$ | $a = 0.985$, $b = 0.676$ $c = -27.419$ | $\dot{X}(t) = 1.062X_i + \sum_{j=1}^{N} A_{ij} \frac{-0.359X_i}{0.173X_i - X_j}$ |
| Spain | $a = 0.361$, $b = 5.163$ | $a = 1.093, b = 1.193$ $c_0 = 2.382, c_1 = 0.884$ $c_2 = 4.275, c_3 = 5.948$ | $a = 0.983$, $b = 2.761$ $c = -2.742$ | $\dot{X}(t) = 1.067X_i + \sum_{j=1}^{N} A_{ij} (0.241 + 0.003X_i - 0.009X_j)$ |

## B Pedestrian dynamics

For the crowd trajectory dataset, we utilize an experimental dataset from a study on pedestrian dynamics, specifically focusing on unidirectional flow in a corridor, where a group of individuals moves through the corridor in the same direction [22]. In the pedestrian dynamic dataset, the network structure is dynamic in the actual situation and has the characteristics of evolving over time. In fact, our approach also can handle both static and dynamic network structures, i.e., the topology A can change over time, represented as $A_{i,j}(t)$.

$$\dot{X}_i(t) = Q_i^{(self)}(X_i) + \sum_{j=1}^{N} A_{i,j}(t) Q_{i,j}^{(inter)}(X_i, X_j), \quad A_{i,j}(t) = \begin{cases} 1, \text{if } j \in \mathcal{N}(i) \\ 0, \text{otherwise} \end{cases}$$

The specific neighbor selection relation: For node $i$ , select the $K$ nodes with its smallest value as neighbors. The mathematical expression is as follows:

$$\mathcal{N}_i(t) = \left\{ j | j \in \text{argtop}_{j \neq i}^{K}(-d(X_i(t), X_j(t))) \right\}, \quad d(X_i(t), X_j(t)) = ||X_i(t) - X_j(t)||_2$$

Here, argtop$^K$ denotes the node index that takes the first $K$ minimum distance, as shown in the specific Supplementary Figure 16. Given that we know $X_i(t)$ at any time $t$, i.e., the positions of each person, we can dynamically construct $A_{i,j}(t)$ based on this positions when inferring the governing equation. Then, by utilizing the signal decoupling module, we can separate the self dynamics and the interaction dynamics using the previously mentioned prior template. Finally, we employ symbol regression to identify the corresponding white-box expressions.

Specifically, the experimental data were collected at a fixed time interval of 0.04 seconds and established the training duration $T$ as 6. The center of the corridor was designated as the coordinate origin, resulting in a range for X of $[-5, 5]$, where -5 denotes the starting point and 5 indicates the destination. In this work, 17 participants commenced their journey from the initial point towards the destination. During the testing phase, we selected trajectories from all participants over the last 30 time steps to serve as testing set.

# XI Pseudo-code of the LLC

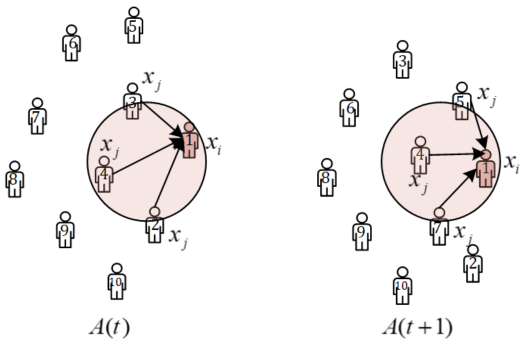


Supplementary Figure 16: Schematic diagram of dynamic topology for K-nearest neighbor construction network. The value of K is 3.

---

**Algorithm 1** Pseudo-code of LLC

---

**Require:**
- $X \in \mathbb{R}^{T \times N \times d}$: System state tensor
- $A \in \{0,1\}^{N \times N}$: Network adjacency matrix
- $M_x \in \{0,1\}^{T \times N \times d}$: State observation mask
- $M_a \in \{0,1\}^{N \times N}$: Topology mask

**Ensure:**
- $Q_i^{(\text{self})}$: Symbolic self-dynamics expressions
- $Q_{ij}^{(\text{inter})}$: Symbolic interaction dynamics expressions

**Data Preprocessing:**
$\delta t \leftarrow \text{IntervalSelect}(X)$ ▷ Identify the optimal time interval through an iterative process that incorporates simulated annealing to achieve accurate signal decoupling
$X, \hat{X} \leftarrow \text{FiniteDifference}(X, \delta t)$ ▷ Five point finite difference as Eq.(2)
`sparse_A` $\leftarrow \text{Sparse}(A)$ ▷ The sparse representation of the topological structure A
**Signal Decoupling:**
$\hat{Q}_i^{(\text{self})} \leftarrow \psi^f(X_i(t))$ ▷ Self-dynamics fitting
$\hat{Q}_{ij}^{(\text{inter})} \leftarrow \psi^{g_0}(X_i, X_j) + \psi^{g_1}(X_i)\psi^{g_2}(X_j)$ ▷ Interaction dynamics fitting
$\sum \hat{Q}_{i,j}^{(\text{inter})} \leftarrow \text{ScatterSum}(\hat{Q}^{inter}, \mathcal{N}(i), \text{dim\_size} = N)$ ▷ Use *ScatterSum* to aggregate neighbor nodes, $\mathcal{N}(i)$ denotes the neighbors of node $i$
$X \leftarrow X \odot M_x$ ▷ $X$ is masked in training
$A \leftarrow A \odot M_a$ ▷ $A$ is masked in training
Train $\psi^f, \psi^{g_0}, \psi^{g_1}, \psi^{g_2}$ with $\mathcal{L}$ in Eq.(5)
**Symbolic Regression:**
Input: $\hat{Q}^{(\text{self})}$: Well-fitted self dynamics; $\hat{Q}^{(\text{inter})}$: Well-fitted interaction dynamics;
$(X_{\text{sub}}, \widehat{Q}_{\text{sub}}^{(\text{self})}) \leftarrow \text{KMeansSubsample}(X, \widehat{Q}^{(\text{self})})$ ▷ KMeans sampling is performed on self dynamics
$Q_i^{(\text{self})} \leftarrow \text{SymbolicRegression}(X_{\text{sub}}, \widehat{Q}_{\text{sub}}^{\text{self}})$ ▷ Self-dynamics expressions effectively generate through a pre-trained symbolic regression method
$(\xi_{ij}^{\text{sub}}, \widehat{Q}_{\text{sub}}^{\text{inter}}) \leftarrow \text{KMeansSubsample}(\xi_{ij}, \widehat{Q}^{(\text{inter})})$ ▷ KMeans sampling is performed on interaction dynamics, $\xi_{ij}$ donates $(X_i, X_j)$
$Q_{i,j}^{(\text{inter})} \leftarrow \text{SymbolicRegression}(\xi_{ij}^{\text{sub}}, \hat{Q}_{\text{sub}}^{\text{inter}})$ ▷ Interaction dynamics expressions effectively generate through a pre-trained symbolic regression method
**return** $Q_i^{(\text{self})}, Q_{i,j}^{(\text{inter})}$ ▷ Assess whether additional data is required to support the equation, if so, restart **Data Preprocessing;** otherwise, return

---

# References


[1] Voit, E.O.: Computational Analysis of Biochemical Systems: a Practical Guide for Biochemists and Molecular Biologists. Cambridge University Press, Cambridge University (2000)

[2] Mazur, J., Ritter, D., Reinelt, G., Kaderali, L.: Reconstructing nonlinear dynamic models of gene regulation using stochastic sampling. BMC bioinformatics **10**, 1–12 (2009)

[3] Karlebach, G., Shamir, R.: Modelling and analysis of gene regulatory networks. Nature reviews Molecular cell biology **9**(10), 770–780 (2008)

[4] Gao, J., Barzel, B., Barabási, A.-L.: Universal resilience patterns in complex networks. Nature **530**(7590), 307–312 (2016)

[5] MacArthur, R.: Species packing and competitive equilibrium for many species. Theoretical population biology **1**(1), 1–11 (1970)

[6] Wilson, H.R., Cowan, J.D.: Excitatory and inhibitory interactions in localized populations of model neurons. Biophysical journal **12**(1), 1–24 (1972)

[7] Pastor-Satorras, R., Castellano, C., Van Mieghem, P., Vespignani, A.: Epidemic processes in complex networks. Reviews of modern physics **87**(3), 925–979 (2015)

[8] Pietras, B., Daffertshofer, A.: Network dynamics of coupled oscillators and phase reduction techniques. Phys. Rep. **819**, 1–105 (2019)

[9] Chen, Y., Kolokolnikov, T.: A minimal model of predator-swarm dynamics. Journal of the Royal Society Interface **11**, 20131208 (2014)

[10] ERDdS, P., R&wi, A.: On random graphs i. Publ. math. debrecen **6**(290-297), 18 (1959)

[11] Barabási, A.-L., Albert, R.: Emergence of scaling in random networks. science **286**(5439), 509–512 (1999)

[12] Yan, G., Vértes, P.E., Towlson, E.K., Chew, Y.L., Walker, D.S., Schafer, W.R., Barabási, A.-L.: Network control principles predict neuron function in the caenorhabditis elegans connectome. Nature **550**(7677), 519–523 (2017)

[13] Scheffer, L.K., Xu, C.S., Januszewski, M., Lu, Z., Takemura, S.-y., Hayworth, K.J., Huang, G.B., Shinomiya, K., Maitlin-Shepard, J., Berg, S., *et al.*: A connectome and analysis of the adult drosophila central brain. elife **9**, 57443 (2020)

[14] Baggio, G., Bassett, D.S., Pasqualetti, F.: Data-driven control of complex networks. Nat. Commun. **12**(1), 1429 (2021)

[15] Sase, T., Ramírez, J.P., Kitajo, K., Aihara, K., Hirata, Y.: Estimating the level of dynamical noise in time series by using fractal dimensions. Physics Letters A **380**(11-12), 1151–1163 (2016)

[16] Johnson, D.H.: Signal-to-noise ratio. Scholarpedia **1**(12), 2088 (2006)

[17] Majhi, S., Perc, M., Ghosh, D.: Dynamics on higher-order networks: A review. Journal of the Royal Society Interface **19**(188), 20220043 (2022)

[18] Battiston, F., Cencetti, G., Iacopini, I., Latora, V., Lucas, M., Patania, A., Young, J.-G., Petri, G.: Networks beyond pairwise interactions: Structure and dynamics. Phys. Rep. **874**, 1–92 (2020)

[19] FitzHugh, R.: Impulses and physiological states in theoretical models of nerve membrane. Biophysical journal **1**(6), 445–466 (1961)

[20] Moghtadaei, M., Hashemi Golpayegani, M.R.: Complex dynamic behaviors of the complex lorenz system. Scientia Iranica **19**(3), 733–738 (2012) https://doi.org/10.1016/j.scient.2010.11.001

[21] Dong, E., Du, H., Gardner, L.: An interactive web-based dashboard to track covid-19 in real time. The Lancet infectious diseases **20**(5), 533–534 (2020)

[22] Boltes, M., Seyfried, A.: Collecting pedestrian trajectories. Neurocomputing **100**, 127–133 (2013)

[23] OpenFlights: OpenFlights Data. Accessed: 2024-11-04 (2020). https://openflights.org/data.html

[24] Gao, T.T., Yan, G.: Autonomous inference of complex network dynamics from incomplete and noisy data. Nat. Comput. Sci. **2**(3), 160–168 (2022)
